# Vision toolkit part 2. features and metrics for assessing oculomotor signal: a review

**DOI:** 10.3389/fphys.2025.1661026

**Published:** 2025-11-05

**Authors:** Quentin Laborde, Axel Roques, Allan Armougum, Nicolas Vayatis, Ioannis Bargiotas, Laurent Oudre

**Affiliations:** ^1^ Université Paris Saclay, Université Paris Cité, ENS Paris Saclay, CNRS, SSA, INSERM, Centre Borelli, Gif-surYvette, France; ^2^ SNCF, Technologies Department, Innovation & Research, Saint Denis, France; ^3^ Thales AVS France, Training & Simulation, Osny, France; ^4^ Université Paris-Saclay, Inria, CIAMS, Gif-surYvette, France

**Keywords:** segmentation algorithm, oculomotor dynamics, fixations, saccades, smooth-pursuits, signal processing, eye-tracking

## Abstract

Eye movement analysis provides critical insights across domains such as perception, cognition, neurological diagnostics, and human-computer interaction. However, reliable quantification of oculomotor remains challenging due to the lack of clear boundaries between fixations, saccades, and smooth pursuits, or variability across individuals and contexts. This article reviews methods for segmenting oculometry data into canonical oculomotor events, and the computational tools that can be used to characterize them. Binary segmentation employs mostly threshold-based algorithms and learning-based algorithms to distinguish fixations from saccades. Ternary segmentation additionally considers smooth pursuits using primarily threshold-based approaches and deep learning techniques. The common challenges in the practical application of segmentation algorithms are highlighted, namely, parameter sensitivity, noise, and head movement artifacts in mobile eye trackers, and emphasize the need for standardized benchmarks. The usual oculomotor metrics that can be inferred from the canonical movements are described, encompassing temporal, spatial, and kinematic features. The critical insights they provide for cognitive and clinical research in fields such as reading comprehension, neurological disorder diagnostics, and sensorimotor development, are outlined. Finally, relatively underexplored methods from signal processing, including spectral, stochastic, and topological methods, are presented. Their potential in revealing oscillatory patterns and structural complexities in gaze dynamics is detailed. Together, these approaches enhance our understanding of eye movement behavior, with significant implications for psychology, neuroscience, and human-computer interaction.

## 1 Introduction

Eye movement research has a rich history, beginning with foundational work by Dodge and Cline (1901) in the early $20^{th}$ century. Technological advancements have since enhanced the measurement, storage, and analysis of eye movements, enabling significant progress in understanding their underlying mechanisms. The growing accessibility of eye-tracking tools has expanded their use across global research laboratories, fostering specialized subfields like neuroscience, psychology, marketing, and medicine. Each discipline has provided critical insights, collectively shaping modern eye movement research.

A primary goal in eye movement research is to extract metrics that characterize the oculomotor system. Due to their close link with visual attention, eye movements analysis is a powerful tool for studying cognitive and behavioral processes. Recent studies have integrated eye movement analysis into cognitive psychology, exploring areas like language processing, reading, and problem-solving (Rayner, 1998). Research has also investigated connections between eye movements, visual attention, and perception (Collins and Doré-Mazars, 2006; Schütz et al., 2011). Additionally, individual differences in oculomotor patterns have paved the way for eye movement biometrics (Rigas and Komogortsev, 2016).

Clinical research increasingly employs eye movement analysis as a non-invasive method to identify neural irregularities linked to neurodegenerative and neurological disorders (MacAskill and Anderson, 2016). Distinct oculomotor patterns have been observed in individuals with early-stage Alzheimer’s disease (Fernández et al., 2013) and Parkinson’s disease (Wetzel et al., 2011), highlighting their potential as biomarkers for early diagnosis and disease monitoring. Furthermore, a growing body of evidence explores oculomotor features in behavioral disorders such as attention deficit hyperactivity disorder (ADHD) (Fried et al., 2014) and autism spectrum disorder (ASD) (Klin et al., 2002; Shirama et al., 2016), offering valuable insights into the neurocognitive mechanisms underlying these conditions.

The rapid growth of eye movement research has also brought significant challenges. The increasing volume of publications can obscure critical insights, while fragmentation across sub-disciplines hinders effective knowledge integration. As the different research communities pursue distinct objectives, definitions and methodologies often become highly specialized, which limits their generalizability. This has contributed to a fragmented conceptual framework within the field. Notably, a recent study highlights that even fundamental terms such as *fixation* and *saccade* are defined inconsistently, resulting in *conceptual confusion* (Hessels et al., 2018). These definitions vary considerably depending on whether the perspective is functional, oculomotor, or computational, with little consensus even within individual subfields.

Beyond conceptual and terminological inconsistencies, the field lacks standardized methods for defining and extracting eye movement features. Most studies emphasize feature subsets tailored to specific research questions, and the methodological variability in segmenting raw gaze data into canonical movements—such as fixations, saccades, and smooth pursuits—undermines reproducibility. The growing availability of portable, cost-effective eye-tracking devices has facilitated the study of naturalistic behavior in both laboratory and real-world settings (Hayhoe and Ballard, 2005; Land, 2009). However, the absence of standardized analysis protocols limits comparability between studies and hinders the integration of knowledge. This work aims to address these challenges by proposing a unified methodological framework to improve interoperability across research communities and improve comparison across experimental contexts.

This review focuses on methods for segmenting, extracting and analyzing fixations, saccades, and smooth pursuits, building on prior comprehensive reviews of fixation and saccade features (Sharafi et al., 2015; Rigas et al., 2018; Brunyé et al., 2019; Skaramagkas et al., 2021; Mahanama et al., 2022a; Spering, 2022) and pursuit-based features (Skaramagkas et al., 2021; Mahanama et al., 2022a; Spering, 2022). Some reviews target specific domains, such as emotional and cognitive processes (Skaramagkas et al., 2021) or decision-making (Spering, 2022). Additionally, several studies, including Komogortsev et al. (2010b); Birawo and Kasprowski (2022); Startsev and Zemblys (2023), evaluate segmentation algorithms, often comparing their performance on open-source datasets and proposing quality metrics. This work aligns with these efforts by reviewing segmentation methods and their associated oculomotor features.

Specifically, this review surveys methodologies for quantifying oculomotor system activity and explores their diverse applications. While not exhaustive due to the breadth and specialization of some methods, it provides a concise overview of key approaches for characterizing canonical eye movements and their oculometric signals. The following sections are organized as follows. Section 2 introduces segmentation algorithms for classifying fixations, saccades, and smooth pursuits. Two primary analytical approaches are then explored: physiological analysis—Section 3 — which extracts meaningful features like shape, dynamics, and kinematics from segmented sequences, and signal-based analysis—Section 4 — which applies time-series descriptors to examine eye movement behavior from a global dynamic perspective. Although a detailed discussion of metrics is beyond the scope of this review, we aim to provide a unified framework for oculometric signal analysis.

This article is part of a series of four reviews dedicated to methods for analyzing oculomotor signals and gaze trajectories. The overarching goals of the series are to evaluate the application of eye movement and gaze analysis techniques across diverse scientific disciplines and to work toward a unified methodological framework by defining standardized representations and concepts for quantifying eye-tracking data. The first article in the series, already published in *Frontiers in Physiology* (Laborde et al., 2025), provided an overview of current knowledge on canonical eye movements, with particular emphasis on distinguishing findings obtained in controlled laboratory settings from those observed in more natural, head-free conditions.

## 2 Segmentation algorithms

Three archetypal gaze patterns can typically be observed in eye-tracking data: periods of relative stability, rapid eye shifts, and slower shifts corresponding to the tracking of moving objects. These are commonly assumed to reflect the three main canonical oculomotor events that direct gaze movements, namely, fixations, saccades and smooth pursuits. Thus, a necessary preliminary step in eye-movement analysis is often to identify these canonical events from a continuous stream of gaze data using segmentation algorithms. Segmentation algorithms employ a number of predefined criteria, based on the underlying characteristics of the oculomotor events, in order to distinguish them. Such a process aligns with the traditional neurophysiological view, which postulates that distinct neural mechanisms govern specific movement types, such as the superior colliculus for saccades or the cerebellum for smooth pursuits.

However, the organization of the oculomotor system as a discrete set of events has been questioned, notably in the context of natural viewing conditions (Steinman et al., 1990). Under ecological conditions, a richer repertoire of ocular behavior can be observed. This results in potential overlap between the characteristics of the oculomotor events, which makes the segmentation task more challenging. Therefore, it seems more appropriate to refer to segmentation algorithms as event classification rather than event detection, since they merely assign a discrete event type to each data period based on some computationally inferred features—*e.g.*, velocity thresholds for saccades or duration thresholds for fixations. This distinction is critical, as misclassification can distort interpretations of visual attention in fields such as psychology, neuroscience, and human-computer interaction.

A major challenge in eye movement segmentation is the dependence on user-defined parameters, such as velocity thresholds for saccades or minimum fixation durations. Although these events are grounded in physiological phenomena, no theoretical consensus exists on parameter values that definitively distinguish movement types. For instance, the transition from slow movements, such as smooth pursuits or drifts, to rapid saccades lacks a clear, physiologically validated threshold. Studies investigating optimal parameterization for specific algorithms (Blignaut, 2009; Shic et al., 2008) indicate that variations in parameter settings significantly influence classification outcomes (Komogortsev et al., 2010b; Salvucci and Goldberg, 2000). This sensitivity hampers reproducibility and can distort findings in fields requiring precise event classification, such as psychology or human-computer interaction. In psychology, for example, precision in detecting fixations is crucial for analyzing attention strategies, such as in studies on reading or visual information processing (Rayner, 1998). For instance, in experimental paradigms measuring cognitive load, accurate identification of fixations enables reliable quantification of the time spent on specific stimuli, thereby revealing underlying attentional processes (Duchowski and Duchowski, 2017). In human-computer interaction (HCI), precise classification of eye movement events is equally important for evaluating the usability of user interfaces (Jacob and Karn, 2003). Correct detection of saccades and fixations, for example, allows for the identification of interface areas that attract users’ attention or pose accessibility issues, directly influencing the design of more intuitive interfaces.

Conversely, errors in the detection of fixations or saccades can have significant repercussions on the interpretation of data in studies in cognitive psychology and human-computer interaction (HCI). As shown by Duchowski and Duchowski (2017) and Nyström and Holmqvist (2010), erroneous classification of eye movement events can bias the analysis of attentional processes or user behaviors. For example, a fixation incorrectly identified as a saccade can distort measures of cognitive load in experimental paradigms, leading to erroneous conclusions about underlying cognitive mechanisms (Rayner, 1998). Similarly, in HCI, imprecise detection of eye movement events can result in an incorrect evaluation of an interface’s usability, affecting recommendations for its optimization (Jacob and Karn, 2003). As such, threshold-based methods, including velocity or dispersion thresholding, provide computational interpretations of oculomotor events, but their criteria often vary across studies and implementations, leading to inconsistent classifications of identical gaze data due to insufficient standardization, which compromises the reproducibility of results in contexts requiring high precision (Holmqvist et al., 2011).

Finally, researchers must consider the coordinate system used when analyzing eye-tracking data, particularly with mobile eye trackers that permit free head movement. Unlike stationary trackers, which use a head-referenced coordinate system, mobile trackers record gaze in a world-referenced system, where head movements can complicate event classification. To avoid such conceptual confusion, researchers should ensure proper head movement compensation and clearly report their coordinate system. For a detailed discussion of challenges in defining oculomotor events, see the review by Hessels et al. (2018). Note that considerations regarding the utilization and transformation of these coordinates in relation to a moving observer’s visual field are addressed in the first part of this review series (Laborde et al., 2025)

Although some authors have called for the standardization of eye movement classification algorithms and evaluation tools (Komogortsev et al., 2010a), Startsev and Zemblys (2023), there is currently no clear consensus on how to benchmark these methods. This lack of agreement poses challenges to the development and comparison of new segmentation approaches. To address this gap, several concrete proposals have been suggested in the literature. First, minimal reporting standards could be established, requiring authors to clearly specify algorithm parameters, eye-tracker sampling rates, stimulus types, and data preprocessing steps. Second, the use of shared, openly available datasets would enable reproducible evaluation across diverse conditions, including static, dynamic, and naturalistic stimuli. Third, benchmark competitions or challenges could be organized, similar to practices in computer vision and machine learning, where algorithms are tested on identical datasets using standardized metrics such as precision, recall, F1-score, Cohen’s Kappa, and RMSD. By adopting these practices, the field could facilitate more transparent, reproducible, and comparable assessments of eye movement segmentation algorithms, ultimately accelerating methodological improvements.

In this review, we focus on fixations, saccades, and smooth pursuit eye movements, as these are the most commonly studied and well-characterized oculomotor events in the literature. Other canonical eye movement events, such as vergence, optokinetic reflexes, and vestibulo-ocular reflex (VOR), are not included. These events are less frequently analyzed in eye-tracking studies, and their detection often requires specialized experimental setups or instrumentation beyond conventional gaze-tracking paradigms. By concentrating on fixations, saccades, and pursuits, we ensure that the discussion is grounded in well-supported empirical evidence while acknowledging that additional eye movement types remain an important direction for future work. Despite these challenges, the following sections provide an overview of widely used segmentation methods (Salvucci and Goldberg, 2000; Komogortsev and Karpov, 2013; Andersson et al., 2016).

### 2.1 Separating saccades from fixations

**FIGURE 1:**
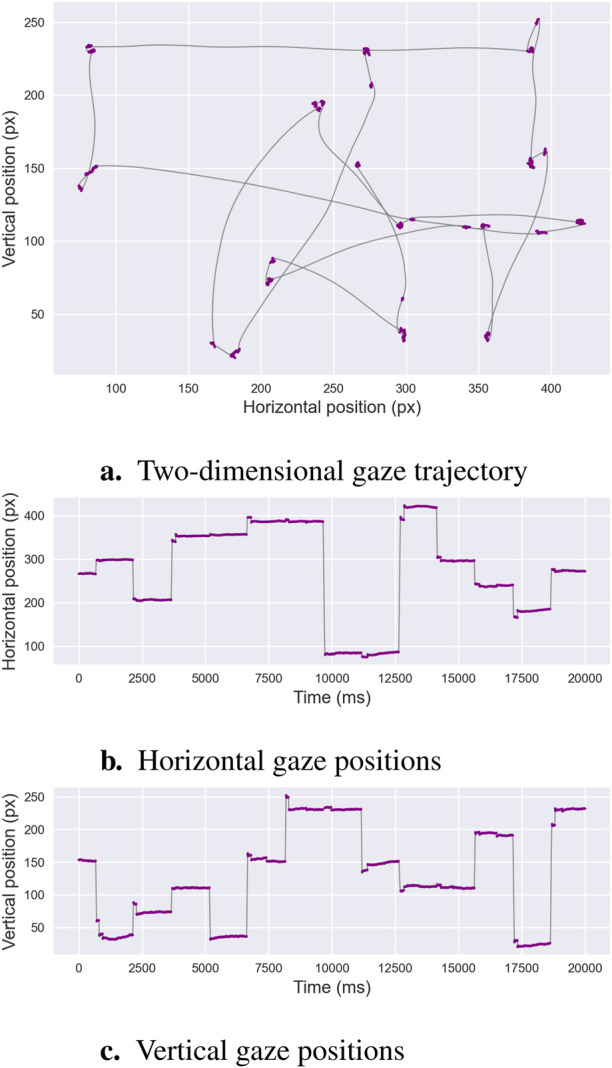
Binary Segmentation. This example illustrates an oculomotor recording containing both fixations and saccades. Panel **(a)** depicts the two-dimensional gaze trajectory, with alternating periods of stability—fixations shown in purple—and rapid ballistic reorientations—saccades shown in gray. Panels **(b,c)** present the horizontal and vertical gaze positions over time, respectively, using the same color scheme. These characteristic patterns form the basis of *binary segmentation algorithms*, which aim to distinguish fixation sequences from saccadic sequences.

Numerous algorithms have been developed to address the challenge of distinguishing saccades from fixations, a process known as binary segmentation. This is illustrated in Figure 1, which depicts alternating periods of relative gaze stability—fixations, marked in purple—and rapid gaze reorientations—saccadic eye movements. The recording shown in Figure 1 is of exceptionally high quality, with minimal noise or signal loss. In contrast, real-world eye-tracking data often exhibit lower quality due to several factors. For instance, blinks or partial eyelid closures interrupt the signal, while head movements or poor participant stabilization can introduce spatial jitter. Changes in lighting conditions or reflections on glasses can reduce the accuracy of gaze detection, and low sampling rates or occasional data dropouts may cause missing or irregular samples. Additionally, physiological variability, such as micro-saccades or pupil size fluctuations, can further complicate event classification. These factors collectively increase the difficulty of distinguishing fixations from saccades, emphasizing the need for robust segmentation algorithms that can tolerate noise and handle incomplete or variable-quality data.

Binary segmentation algorithms are broadly categorized into *threshold-based* and *learning-based* approaches. Threshold-based methods rely on predefined computational criteria, such as velocity or spatial dispersion, to classify fixations and saccades, ensuring transparent, rule-based classification. In contrast, learning-based methods, encompassing machine learning and deep learning techniques, infer patterns from annotated training data, which reflect expert or task-specific interpretations of fixations and saccades. These annotations may reduce the transparency of classification criteria compared to threshold-based methods due to their reliance on subjective or context-dependent definitions.

#### 2.1.1 Threshold-based algorithms

The velocity-threshold identification (I-VT) algorithm (Salvucci and Goldberg, 2000) is a widely adopted method for distinguishing fixations from saccades in eye movement data. It leverages the distinct velocity profiles of eye movements: low velocities characterize fixations, while high velocities indicate saccades. The I-VT algorithm calculates the absolute velocity between consecutive gaze samples and classifies each sample as a fixation or saccade based on a user-defined velocity threshold. To address the subjectivity of manual threshold selection, Nyström and Holmqvist (2010) proposed an adaptive I-VT variant that dynamically computes thresholds for peak velocities and saccade onset/offset detection based on statistical properties of the data. This method incorporates constraints derived from the physical characteristics of eye movements—such as minimum and maximum velocities, accelerations, and event durations—to filter noise and enhance classification accuracy.

In contrast to velocity-based methods, the dispersion-threshold identification (I-DiT) algorithm offers an alternative approach by leveraging the tendency of fixation points—characterized by relatively low velocity—to cluster spatially (Salvucci and Goldberg, 2000; Komogortsev et al., 2010a; Andersson et al., 2016). The I-DiT algorithm distinguishes fixations from saccades based on the spatial dispersion of consecutive gaze points within a defined temporal window. Dispersion is quantified by summing the ranges—*i.e.*, the differences between the maximum and minimum values—of the gaze coordinates in both the horizontal and vertical dimensions. If the resulting dispersion value falls below a predefined threshold, the corresponding gaze points are classified as a fixation. Otherwise, if the dispersion exceeds the threshold, the sequence is identified as a saccade.

Another notable approach is the minimum spanning tree (MST)-based method (Goldberg and Schryver, 1995; Salvucci and Goldberg, 2000; Komogortsev et al., 2010a; Andersson et al., 2016), which also employs a dispersion-based strategy to evaluate local gaze dispersion within a temporal window of eye position data. Unlike traditional methods, MST-based algorithms model gaze points as nodes in a graph, with edges weighted by the Euclidean distance between corresponding positions. A minimum spanning tree is constructed—typically using Prim’s algorithm (Camerini et al., 1988) — to connect all nodes while minimizing total edge length. The identification by minimum spanning tree (I-MST) algorithm classifies gaze points by applying edge-distance thresholds: points connected by edges shorter than the threshold are grouped as fixation components, while those separated by longer edges are classified as saccadic components. Thresholds may be applied globally across the graph (Komogortsev et al., 2010a) or adapted locally based on vertex density (Goldberg and Schryver, 1995). The MST-based approach offers flexibility, adapts to local data structures, and demonstrates robustness in handling missing or noisy data, making it suitable for complex eye-tracking datasets.

The Density-Threshold Identification (I-DeT) algorithm is an adaptation of the DBSCAN clustering method (Ester et al., 1996). I-DeT extends DBSCAN by incorporating the temporal dimension of gaze data, ensuring that segmented events reflect the sequential nature of eye movements. As introduced by Li et al. (2016), a gaze point is classified as a core point if: (i) at least a minimum number of gaze points lie within a specified spatial radius of the reference point, forming a local neighborhood; and (ii) these neighboring points form a temporally contiguous sequence in the gaze dataset. Fixations are identified as clusters comprising core points and their associated neighborhoods, while non-core, non-neighbor points are classified as saccades or noise. This integration of spatial and temporal constraints makes I-DeT robust for segmenting gaze data, though its performance depends on careful parameter tuning to avoid over—or under—segmentation.

Building on classical signal processing, Kalman filter-based algorithms (I-KF) model eye movements as a dynamic system. The two-state Kalman filter, as proposed by Komogortsev and Khan (2007), represents eye movements using position and velocity states, assuming linear dynamics and Gaussian noise. The algorithm operates recursively in two phases: (i) the predict phase, which forecasts the next state based on the system model, and (ii) the update phase, which refines the prediction using observed data to produce a more accurate state estimate. Saccade detection employs a Chi-square test (Sauter et al., 1991) to assess discrepancies between predicted and observed gaze velocities, with a threshold determining whether a sample is classified as a saccade—high velocity—or fixation—low velocity. This approach excels in handling noisy data by combining predictive modeling with statistical testing, offering a robust framework for eye movement classification applicable in fields such as human-computer interaction and clinical research.

#### 2.1.2 Learning-based algorithms

The Hidden Markov Model Identification (I-HMM) algorithm, introduced by Salvucci and Goldberg (2000), extends the velocity-threshold identification (I-VT) approach by employing a probabilistic framework to segment eye movements into fixations and saccades. I-HMM models eye movements as a sequence of two latent states—fixation and saccade—each characterized by a Gaussian velocity distribution. Fixations typically exhibit low mean velocity, while saccades are defined by high mean velocity—*e.g.*, >200 degrees per second. Transitions between these states are modeled as a first-order Markov process, capturing the temporal dependencies inherent in gaze data. The approach leverages the Baum-Welch algorithm (Bilmes et al., 1998) to estimate model parameters, including state transition probabilities and emission distribution parameters—*e.g.*, mean and variance of velocity distributions—from training data. Subsequently, the Viterbi algorithm infers the optimal sequence of states for a given gaze dataset. Unlike deterministic threshold-based methods like I-VT, I-HMM accounts for noise and sequential patterns, providing robust segmentation that is particularly effective for noisy or complex eye-tracking datasets.

The Two-Means Clustering Identification (I2MC) algorithm, introduced by Hessels et al. (2017), is designed to extract fixations from gaze data with high noise levels, such as those recorded from infants. The algorithm employs two-means clustering—k-means with k=2 — on a fixed-length temporal window—typically 200–400 milliseconds—to partition gaze samples into stable—fixation—and rapid—saccade—clusters based on their spatial coordinates. For each window, the number of transitions between clusters is calculated, and each gaze sample is assigned a weight inversely proportional to the number of transitions, reflecting the stability of the cluster assignment. To enhance robustness to noise, this process is applied across multiple down-sampled versions of the gaze signal. The clustering weights for each gaze sample are aggregated and averaged to generate a weight signal, which is then thresholded using an empirically determined cut-off to identify fixation periods, effectively distinguishing fixations from ballistic saccades. I2MC demonstrates robustness to data loss—*e.g.*, due to blinks or tracker errors—and was shown to outperform seven state-of-the-art algorithms on noisy infant data, making it well-suited for applications in developmental psychology, clinical research, and longitudinal studies with variable data quality (Hessels et al., 2017).

Building upon established machine learning techniques, Zemblys et al. (2018) introduced the Random Forest Classifier (I-RF) algorithm to distinguish fixations, saccades, and potentially other eye movement events from raw gaze data. The I-RF model is trained on a set of 14 features, including spatial measures—*e.g.*, root mean square of sample-to-sample displacement, standard deviation of gaze positions, bivariate contour ellipse area—and statistical measures—*e.g.*, sample dispersion, kurtosis. The random forest classifier leverages these features to model complex, non-linear relationships, achieving high classification accuracy. However, a key limitation is the reliance on hand-tagged training data, which is labor-intensive and hinders scalability. Reproducibility is also challenging, as model performance depends on the quality and representativeness of training datasets. Additional limitations include the computational cost of feature extraction and the risk of overfitting to specific datasets. Nevertheless, I-RF is particularly valuable in eye-tracking research for applications in cognitive psychology, human-computer interaction, and clinical diagnostics, offering robustness to noise and the potential to detect diverse eye movement types when trained appropriately.

The evaluation of binary segmentation algorithms, which aim to distinguish fixations from saccades, has been reported in benchmark studies comparing algorithm outputs to human coders using high-frequency datasets that include static images, text, moving dots, and videos (Andersson et al., 2016). These studies provide a valuable baseline for assessing segmentation quality. Performances are generally summarized using metrics such as Cohen’s Kappa, which captures agreement with human annotations, or RMSD for event durations, which reflects temporal precision. However, reported values vary considerably depending on the dataset, the type of stimulus, and the specific evaluation protocol, making it difficult to directly compare results across studies.

Among threshold-based methods, the velocity-threshold approach (I-VT) typically reaches Kappa values around 0.65−−0.75 for static image datasets but drops markedly in dynamic conditions, particularly for fixations (Andersson et al., 2016). The dispersion-based algorithm (I-DiT) rarely exceeds 0.45 and shows high sensitivity to noise, while I-MST adapts better to missing data but yields modest agreement overall, usually between 0.3 and 0.5 (Andersson et al., 2016). Kalman filter approaches (I-KF) report reasonable performance for saccades—up to 0.6 — but poor fixation detection. More recently, density-based methods such as I-DeT, inspired by clustering techniques, have been proposed as more robust under noise and data loss, though systematic benchmarks remain scarce (Li et al., 2016).

Learning-based approaches tend to report more robust and generalizable performance, particularly in challenging or noisy datasets. Hidden Markov models (I-HMM) achieve balanced results across stimulus types, with Kappa values close to 0.7 for saccades (Andersson et al., 2016). The two-means clustering method (I2MC), developed specifically for noisy infant recordings, reports an average F1-score of 0.83 across seven independent datasets, consistently outperforming several threshold-based methods (Hessels et al., 2017). Random forest classifiers (I-RF) have achieved state-of-the-art sample-level results, with F1-scores near 0.97 and Kappa values around 0.85 in validation data, though performance decreases to about 0.70 on independent test sets (Zemblys et al., 2018).

In summary, threshold-based methods are attractive for their simplicity and efficiency and remain effective under controlled static conditions, but they degrade substantially in noisy or dynamic environments. Learning-based methods demonstrate greater resilience, adaptability, and the ability to model complex data patterns, although they require annotated training datasets and greater computational resources. It is important to emphasize that these are reported performances drawn from heterogeneous studies, and differences in dataset characteristics, sampling frequency, and evaluation protocols likely account for a substantial part of the observed variability across algorithms.

### 2.2 Separating smooth pursuits from fixations and saccades

**FIGURE 2:**
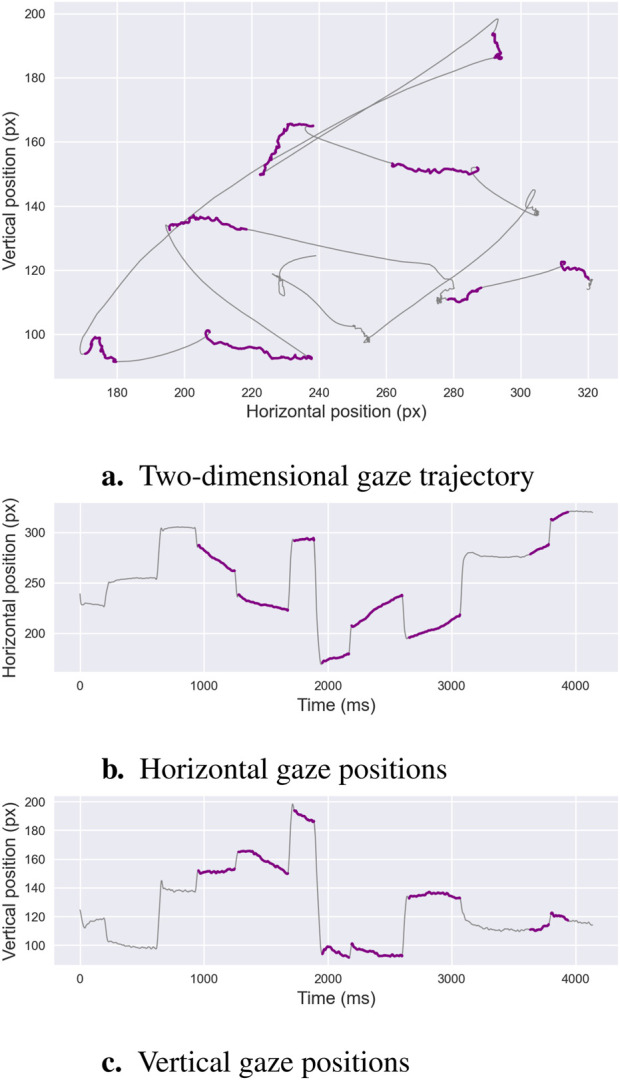
Ternary Segmentation. This example illustrates an oculomotor recording comprising fixations, saccades, and smooth pursuits. Panel **(a)**shows the two-dimensional gaze trajectory, where fixations are marked in purple, saccades in gray, and smooth pursuits in blue. Panels **(b,c)**display the corresponding horizontal and vertical gaze positions over time, highlighting the gradual directional displacements characteristic of smooth pursuit movements. These distinguishing features are the focus of *ternary segmentation algorithms*, which aim to isolate pursuit sequences from other phases.

The detection of smooth pursuit events, characterized by low-velocity, consistent-directionality eye movements that track moving targets, has received less attention compared to saccade and fixation classification. This task, known as *ternary segmentation*—classifying fixations, saccades, and smooth pursuits—is illustrated in Figure 2, which depicts smooth pursuits—marked in purple—alongside fixations and saccades in high-quality eye-tracking data. Methods for identifying smooth pursuits are broadly categorized into threshold-based and learning-based approaches. Both approaches encounter the same limitations outlined in Section 2.1, including sensitivity to predefined thresholds in threshold-based methods and reliance on annotated training datasets in learning-based methods, which can be labor-intensive and specific to the dataset. Smooth pursuit detection is particularly challenging in noisy or low-quality data—*e.g.*, from low-frequency eye trackers or studies involving infants—often necessitating preprocessing steps such as noise filtering or blink removal to improve accuracy.

#### 2.2.1 Threshold-based algorithms

Typically, a simple velocity threshold is first applied to isolate saccadic events, followed by a second step to distinguish between the remaining movements, namely, *fixation* and *pursuit* events. A straightforward but effective method for this task, known as the I-VVT approach, was proposed by Komogortsev and Karpov (2013). This method builds upon the I-VT algorithm by introducing a second velocity threshold to specifically isolate fixation events. Any remaining data points are then classified as pursuit events. However, a potential limitation of this approach is that eye movement velocities can vary between individuals and even within the same individual depending on the specific task being performed. As such, establishing universally effective thresholds to differentiate smooth pursuits from fixations—both of which are low-velocity movements—presents a challenge. This variability can complicate the application of this algorithm in real-world scenarios, particularly those involving dynamic scenes (Kasneci et al., 2015).

To reduce reliance on velocity thresholds, Komogortsev and Karpov (2013) proposed to distinguish between pursuit and fixation movements using a dispersion threshold combined with a temporal window—an approach commonly referred to as I-VDT. This method naturally extends the I-DiT approach by isolating fixation samples based on their spatial proximity. Similarly, Lopez (2009) proposed an alternative strategy where the standard deviation of movement direction within a time window is used to differentiate between fixation and pursuit events. This approach provides an additional method for segmentation that focuses on directional variability rather than relying solely on velocity-based thresholds.

The Velocity and Movement Pattern Identification (I-VMP) algorithm, proposed by Lopez (2009), provides an advanced method for detecting smooth pursuits in eye-tracking data. I-VMP employs a two-stage approach: it first applies a velocity threshold to isolate saccades, then analyzes the angular displacement between consecutive gaze points to identify smooth pursuits among low-velocity movements. Specifically, the angle between the horizontal axis and the line connecting successive gaze points is projected onto a unit circle, and a Rayleigh score is computed to quantify directional consistency within a defined temporal window. High Rayleigh scores indicate stable directionality, characteristic of smooth pursuits, distinguishing them from fixations, which exhibit random or minimal directional changes. While this method reduces dependence on velocity thresholds compared to traditional approaches, it requires preprocessing steps, such as noise filtering and blink removal, and knowledge of stimulus motion for optimal performance.

Finally, Santini et al. (2016) introduced a Bayesian decision theory-based approach (I-BDT), specifically designed for the classification of smooth pursuit eye movements when viewing dynamic stimuli. Unlike earlier methods that rely on a velocity-based initial step to isolate non-saccadic sequences, this approach directly separates smooth pursuits from saccades and fixations without the need for an initial velocity threshold. Grounded in physiological hypotheses, the I-BDT approach incorporates explicit formulas to compute the likelihoods and priors for each type of eye movement—fixation, saccade, and smooth pursuit. These formulas enable the efficient classification of eye movement events by applying Bayes’ theorem, offering a probabilistic framework for distinguishing between different types of oculomotor behavior.

#### 2.2.2 Learning-based algorithms

Fuhl et al. (2018) introduced the Histogram of Oriented Velocities (I-HOV) method, which adapts a computer vision technique to classify fixations, saccades, and smooth pursuits in eye-tracking data. The I-HOV algorithm computes velocity-weighted angles between a gaze point and its predecessors or successors within a defined temporal window, generating a histogram that serves as a meta-representation of local gaze behavior for each sample. These histograms are used as feature vectors for machine learning algorithms, such as random forests, k-nearest neighbors, and support vector machines, to classify eye movement types. Similar to the I-VMP algorithm (Lopez, 2009), I-HOV leverages the consistent directionality and low-velocity profiles of smooth pursuits to distinguish them from fixations and saccades. While effective for ternary segmentation, I-HOV relies on high-quality annotated training data and is computationally intensive. Its performance is also sensitive to noise and the limitations of low-frequency eye trackers, which may reduce the accuracy of velocity and angle calculations.

Recent advances in eye movement classification have leveraged deep learning techniques to distinguish smooth pursuit sequences from fixations and saccades. One such approach, proposed by Hoppe and Bulling (2016), employs a convolutional neural network (CNN) combined with data windowing. In this method, gaze points within each temporal window are transformed into the frequency domain using a Fourier transform and then input to the CNN, which classifies the eye movement type. Similarly, Fuhl et al. (2021) introduced a CNN-based method, termed I-CNN, that operates directly on windowed raw eye data to isolate oculomotor events. These deep learning approaches demonstrate significant effectiveness, particularly when trained on datasets tailored to specific experimental conditions and eye-tracking devices, underscoring their potential for robust eye movement classification. However, their performance remains heavily dependent on the quality and annotation of training data, which can substantially impact model accuracy and generalizability.

Ternary segmentation, tasked with classifying fixations, saccades, and smooth pursuits, presents greater challenges than binary segmentation due to the subtle low-velocity characteristics of smooth pursuits. Insights from Komogortsev and Karpov (2013), Santini et al. (2016), Fuhl et al. (2018), and Fuhl et al. (2021), evaluated on varied datasets with dynamic stimuli, provide a foundation for assessing performance, although quantitative benchmarks remain less comprehensive than for binary segmentation. Moreover, the different evaluations were conducted on distinct datasets, making it challenging to provide a reliable comparative analysis of the various segmentation methods. As such, the following paragraphs will focus on qualitative considerations.

Among threshold-based approaches, extensions of velocity- and dispersion-threshold methods—*e.g.*, I-VVT, I-VDT—have been applied to pursuits, while variants such as I-VMP incorporate directional information to reduce velocity ambiguities. Bayesian decision theory (I-BDT) has been reported to outperform dispersion-based methods (I-VDT) on several dynamic datasets at 30Hz , leveraging priors to enhance pursuit detection (Santini et al., 2016). Learning-based methods show greater adaptability. Histogram-based classification (I-HOV) and convolutional neural networks (I-CNN) have been reported to provide robust detection of pursuits in noisy or low-resolution dynamic data, outperforming threshold-based methods in these contexts (Fuhl et al., 2018; 2021).

In summary, ternary segmentation highlights the intrinsic difficulty of reliably detecting smooth pursuits, particularly at low velocities where they overlap with fixations. Threshold-based methods capture faster pursuits but remain sensitive to noise and sampling rate. Bayesian and direction-based extensions have been reported to reduce some of these ambiguities, though results vary across datasets. Learning-based methods appear more promising for handling complex or noisy recordings, especially with CNNs and histogram-based approaches, yet their effectiveness still depends on the availability of well-annotated training corpora. Reported performances point to relative strengths of each family of methods, but the absence of standardized benchmarks makes it difficult to establish a consensus hierarchy of algorithms.

## 3 Physiological features

Applying the segmentation algorithms presented in Section 2 produces a sequence of fixations, saccades, and possibly smooth pursuits from raw gaze data. The following sections will review the most common metrics found in the literature to describe and analyze these oculomotor events.

The fundamental features and metrics for fixations, saccades, and smooth pursuits are summarized in Tables 1–3, respectively. The tables provide a concise description of each feature and references from the literature that offer guidance for their implementation.

**TABLE 1:** Fixation-based features.

Feature name	Description	References
Count	Given a set of fixation sequences, computes the number of fixations	Rigas et al. (2018)
Frequency	Given a set of fixation sequences, computes the number of fixations occurring per second	Rigas et al. (2018)
Duration	Given a fixation sequence, computes the duration of the sequence	Rigas et al. (2018)
First duration	Given a set of fixation sequences, computes the duration of the first fixation sequence identified	Inhoff et al. (2000)
Centroid	Given a fixation sequence, computes centroid position by averaging coordinates of data samples	Rigas et al. (2018)
Drift displacement	Given a fixation sequence, computes the distance between the starting and ending points of the sequence	Rigas et al. (2018)
Drift distance	Given a fixation sequence, computes the sum of distances between each data sample within this sequence	Rigas et al. (2018)
Mean velocity	Given a fixation sequence, computes the mean velocity of data sample within this sequence	Rigas et al. (2018)
Drift velocity	Given a fixation sequence, computes the drift displacement normalized by the fixation duration	Rigas et al. (2018)
BCEA	Given a fixation sequence, computes the bivariate contour ellipse area (BCEA) as the area of the elliptical contour that encompasses a given percentage of sample points of the sequence	Crossland et al. (2004)

**TABLE 2:** Saccade-based features.

Feature name	Description	References
Duration	Given a saccade sequence, computes the duration of the sequence	Rigas et al. (2018)
Frequency	Given a set of saccade sequences, computes the number of saccades occurring per second	Rigas et al. (2018)
Amplitude	Given a saccade sequence, computes the distance between the starting and ending points of the sequence	Rigas et al. (2018)
Travel distance	Given a saccade sequence, computes the sum of distances between each data sample of the sequence	Rigas et al. (2018)
Efficiency	Given a saccade sequence, computes the ratio of saccadic amplitude over the distance traveled	Rigas et al. (2018)
Direction	Given a saccade sequence, computes the deviation from the horizontal plane of the line connecting the start and end points of the sequence	Foulsham et al. (2008)
Successive deviation	Given a set of saccade sequences, computes the angle formed by successive saccadic trajectories, where each saccade is modeled as a vector connecting its start and end points	Foulsham et al. (2008)
Initial direction	Given a saccade sequence, computes the initial direction of the saccadic trajectory after a fixed number of data measures	Ludwig and Gilchrist (2002)
Initial deviation	Given a saccade sequence, computes the angle between the overall direction determined at the endpoint of the saccade, and the initial direction after a fixed number of data measures	Ludwig and Gilchrist (2002)
Maximum curvature	Given a saccade sequence, computes the maximum perpendicular distance from any point along the saccadic trajectory to the straight line connecting the start and end points of the saccade	Ludwig and Gilchrist (2002)
Area curvature	Given a saccade sequence, computes the area under the curve of the sampled saccadic trajectory, relative to the straight-line distance between the saccade starting and ending points	Ludwig and Gilchrist (2002)
Mean velocity	Given a saccade sequence, computes the mean velocity of data samples within the sequence	Rigas et al. (2018)
Peak velocity	Given a saccade sequence, computes the peak velocity of data samples belonging to the sequence	Rigas et al. (2018)
Acceleration profile	Given a saccade sequence, computes the mean acceleration of data sample within the sequence	Rigas et al. (2018)
Mean acceleration	Given a saccade sequence, computes the mean absolute acceleration during the acceleration phase of the saccade, measured from the start point to the timestamp of peak acceleration	Rigas et al. (2018)
Skewness exponent	Given a saccade sequence, computes the shape parameter obtained by fitting a gamma function to the sequence velocity profile	Chen et al. (2002)
Amplitude to duration ratio	Given a saccade sequence, computes the sequence amplitude over duration ratio	Rigas et al. (2018)
Peak velocity to amplitude ratio	Given a saccade sequence, computes the sequence peak velocity over amplitude ratio	Rigas et al. (2018)
Peak velocity to duration ratio	Given a saccade sequence, computes the sequence peak velocity over duration ratio	Rigas et al. (2018)
Peak velocity to velocity ratio	Given a saccade sequence, computes the sequence peak velocity over mean velocity ratio	Rigas et al. (2018)
Main sequence	Given a set of saccade sequences, computes slopes of the amplitude/duration curve and the log peak velocity/log amplitude curve	Bahill et al. (1975)
Latency	Given a saccade sequence and a theoretical trajectory, computes the time difference between the onset of the theoretical saccade and the start time of the corresponding saccade	Whelan (2008)
Latency quantiles	Given a set of saccade sequences and a theoretical trajectory, computes the set of saccade latencies, before evaluating quantiles of the latency distribution	Vullings (2018)
Gain	Given a saccade sequence and a theoretical trajectory, computes the ratio between saccade and target amplitudes	Holmqvist et al. (2011)

**TABLE 3:** Pursuit-based features.

Feature name	Description	References
Duration	Given a pursuit sequence, computes the duration of the sequence	Murray et al. (2020)
Frequency	Given a set of pursuit sequences, computes the number of pursuits occurring per second	Murray et al. (2020)
Amplitude	Given a pursuit sequence, computes the distance between the starting and ending points of the sequence	Mahanama et al. (2022a)
Direction	Given a pursuit sequence, computes the deviation from the horizontal plane of the line connecting the start and end points of the sequence	Rottach et al. (1996)
Mean velocity	Given a pursuit sequence, computes the mean velocity of data sample within the sequence	Mahanama et al. (2022b)
Peak velocity	Given a pursuit sequence, computes the peak velocity of data samples	Mahanama et al. (2022b)
Latency	Given a pursuit sequence and a theoretical trajectory, computes the time difference between the onset of the theoretical smooth pursuit and the start time of the corresponding experimental pursuit	Carl and Gellman (1987)
Initial acceleration	Given a pursuit sequence and a theoretical trajectory, computes the mean second-order position derivative of the sequence in a time interval immediately following pursuit onset	Kao and Morrow (1994)
Triangular overall gain	Given a pursuit sequence and a triangular theoretical trajectory, computes the ratio between pursuit sequence and target mean velocities	Rashbass (1961)
Sinusoidal overall gain	Given a pursuit sequence and a sinusoidal theoretical trajectory, computes the ratio between pursuit sequence and target mean velocities	O’Driscoll and Callahan (2008)
Sinusoidal gain	Given a pursuit sequence and a theoretical trajectory, fits the eye velocity with a trigonometrical curve, before computing the ratio between the peak velocity of the best fitting curve over the target’s peak velocity	Accardo et al. (1995)
Sinusoidal phase	Given a pursuit sequence and a theoretical trajectory, computes the difference between the phases of the best-fitting velocity curve and the target’s velocity profile	Accardo et al. (1995)
Error entropy	Given a pursuit sequence and a theoretical trajectory, computes the pursuit velocity error series as the difference between the experimental pursuit velocities and theoretical stimulus velocities, before evaluating the approximate entropy of the velocity error series	Pincus et al. (1991)
Cross-correlation	Given a pursuit sequence and a theoretical trajectory, computes normalized cross-correlation between the experimental pursuit velocity and theoretical stimulus velocity signals	Rabiner (1978)

### 3.1 Fixation measures

A fixation is defined as a period during which the gaze is stabilized on a specific spatial location, projecting visual stimuli onto the *fovea centralis*, the retinal region with maximal photoreceptor density and visual acuity. Despite attempts to maintain steady fixation on a stationary target, the eyes exhibit continuous, involuntary micromovements, including microsaccades—rapid, small-amplitude saccades—drifts—slow, curvilinear deviations—and tremors—high-frequency, low-amplitude oscillations. This section examines the quantitative features characterizing fixations, including temporal, positional attributes, and dynamic characteristics. These properties are typically analyzed under head-constrained conditions using high-resolution eye-tracking systems to isolate oculomotor behavior.

#### 3.1.1 Temporal features

*Fixation count* is defined as the total number of fixations within a defined time interval or stimulus region. Despite its simplicity, the fixation count remains a cornerstone metric in eye-tracking research due to its robustness and interpretability. It is frequently employed in exploratory analyses before applying more advanced techniques. Fixation count is widely utilized to assess visual attention allocation to regions of interest (ROIs) in textual or pictorial stimuli (Scheiter and Eitel, 2017), infer the depth and efficiency of visual processing (Jacob and Karn, 2003; Park et al., 2015), and investigate how expertise influences oculomotor behavior in visual tasks (Schoonahd et al., 1973; Megaw and Richardson, 1979).

Pioneering work by Goldberg and Kotval (1999) highlighted that a higher number of fixations directed at a stimulus often indicates inefficiency in the search for relevant information. As such, fixation count has been used in eye-tracking studies to identify visual regions that attract more attention or to infer the amount of cognitive effort required for a particular task. For example, in challenging tasks such as source code reading, a higher fixation count could signify increased visual effort and processing time (Binkley et al., 2013; Sharif et al., 2012). The *fixation count* is often expressed per unit of time or relative to a specific task or sub-task. For example, in reading tasks, the *fixation count* can be normalized to the length of the text by dividing the number of fixations by the number of words (Sharafi et al., 2015).

Another critical metric, *fixation duration*, quantifies the temporal dynamics of gaze behavior. Typical fixations last between 200 and 300 milliseconds; however, longer durations on a stimulus may indicate greater processing complexity (Jacob and Karn, 2003; Krejtz et al., 2016b; Liu and Chuang, 2011). This metric is frequently employed in eye-tracking studies to examine complex cognitive functions such as reading comprehension (Raney et al., 2014), learning processes (Liu, 2014), and mental workload assessment (Liu et al., 2022). Furthermore, individual fixation durations may be analyzed independently. A notable example is the *first fixation duration* during reading, which is a commonly reported linguistic measure used to assess initial processing of a word or phrase (Inhoff et al., 2000; Underwood et al., 2000).

The temporal characteristics of eye fixations are often analyzed in relation to specific regions within the visual field that are visually explored. These *areas of interest* (AoI), may represent regions particularly relevant to the task at hand, or with semantical meaning. Under this formalism, fixation duration metrics are also used, albeit with slight variations. For instance, the *dwell time* is defined as the cumulative duration of all fixations during a single visit to an AoI. The *total dwell time* sums all *dwell time* within a specific AoI over the entire experimental session. Additional AoI-specific metrics offer further granularity, such as the *fixation ratio*, defined as the sum of fixation durations within an AoI divided by the total fixation duration across all AoIs, or the *average fixation duration* within an AoI, derived by normalizing the sum of fixation durations by the number of fixations in that AoI. The concept of AoI as a symbolic tool will be explored in greater detail in the *Areas of Interest* part of this review series (Part 4).

#### 3.1.2 Position and drift

The location of visual fixations is widely studied across various contexts, as it is often assumed to reflect the allocation of visual attention (Findlay and Gilchrist, 2003). A robust method for modeling the central position of fixations is the fixation centroid, calculated by averaging the coordinates of gaze points within individual fixation sequences. Analyzing the spatial distribution of these centroids provides valuable insights into the regions of a stimulus that are prioritized during task-specific processing, offering direct evidence of underlying cognitive processes (Henderson, 2003; Rayner, 1998).

For instance, in studies related to face processing, analyses of fixation patterns have identified specific gaze patterns, such as directing attention to a point just below the eyes (Hsiao and Cottrell, 2008; Peterson and Eckstein, 2012). Similarly, in reading tasks, research has shown that both the likelihood of misidentifying a word and the time required for identification decrease when the eyes fixate near the center of the word (O’Regan and Jacobs, 1992; Brysbaert et al., 1996). These phenomena, known as *optimal viewing position* effects, are thought to stem from the rapid decline in visual acuity as retinal eccentricity increases (Nazir et al., 1998).

**FIGURE 3:**
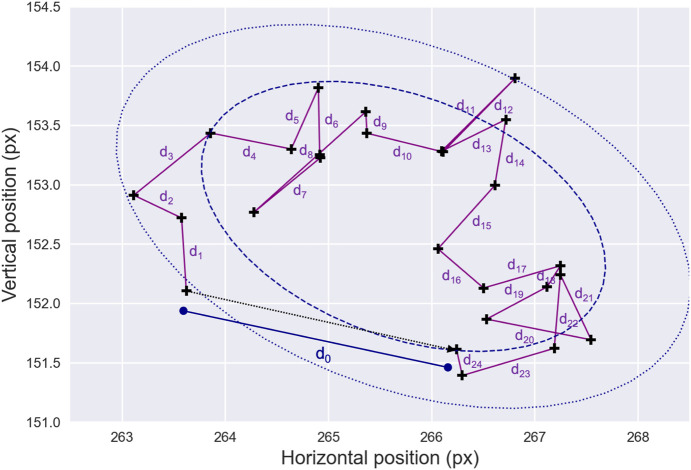
Fixation Drift and Stability. An example of gaze data—black crosses—representing a fixation sequence is shown. Note that the raw data have been largely downsampled for presentation clarity. In this illustration, the drift displacement between the starting and ending points of the fixation sequence is denoted as $d_{0}$ . The cumulative drift distance is computed by summing the distances $d_{1}$ to $d_{24}$ . Additionally, the figure displays the bivariate contour ellipses for probabilities of 0.68 — blue dashed line—and 0.90 —blue dotted line. The areas enclosed by these ellipses are used to compute the BCEA, a commonly used metric for fixation stability.

While fixational sequences typically exhibit limited eye mobility, the variability in the micro-movements can provide valuable information related to oculomotor function. Consequently, several additional features—many of which are illustrated in Figure 3 — have been proposed in the literature to better characterize fixational micro-movements.

As such, the *drift displacement* is calculated as the distance between the starting and ending points of each fixation sequence. Similarly, the *cumulative drift distance*, which reflects ocular stability during fixation, is obtained by summing the distances between all consecutive fixational data samples from a given fixation sequence. Another feature, the *drift mean velocity*, is computed as the average of the first-order position derivatives of the fixation data samples and can be used to characterize the minor movements occurring during fixation sequences. Together, these measures can provide valuable insights into the stability of eye movements during fixation, which may be particularly useful for detecting pathological conditions, such as sight impairments and cerebellar diseases (Leech et al., 1977; Schor and Westall, 1984).

Lastly, fixation stability can be quantified by computing the area of the elliptical contour that encompasses a given percentage of fixation points (Steinman, 1965; Crossland et al., 2004). Assuming that the fixation positions follow a bivariate normal distribution, the dispersion of these positions is represented by an ellipse. The *bivariate contour ellipse area* (BCEA) thus provides a measure of fixation stability, with smaller values indicating more stable fixation. This metric is considered the current *gold standard* to measure the stability of fixation (Crossland et al., 2009) and has been widely used to examine changes in fixational eye movements, particularly in clinical contexts (Shaikh et al., 2016; Montesano et al., 2018; Leonard et al., 2021; Ghasia and Wang, 2022).

### 3.2 Saccade measures

Saccades are rapid, ballistic eye movements that direct the *fovea* toward objects of interest, enabling high-acuity vision. Since the inception of eye movement research, the kinematic properties—*e.g.*, velocity, amplitude—and shape characteristics—*e.g.*, trajectory, curvature—of saccadic eye movements have been extensively studied using diverse measurement techniques, which we will now review and discuss.

In experimental settings, saccadic behavior is investigated using paradigms involving both predictable and unpredictable target conditions. The metrics presented in the following sections are designed to quantify the dynamics of saccadic eye movements in these two conditions, that is free-viewing scenarios and those involving target-based stimuli. These metrics offer critical insights into saccade dynamics and their modulation by experimental manipulations.

#### 3.2.1 Temporal features

*Saccade duration* is a commonly analyzed metric in eye movement research, with typical values ranging from 30 to 70 milliseconds. While these values may vary slightly across studies, various factors have been identified in the literature as influencing saccade duration. For example, during coordinated reaching movements, saccades that accompany hand motions tend to have shorter durations (Donkelaar et al., 2004; Snyder et al., 2002). Conversely, repeated saccades to the same visual stimulus often result in longer durations (Golla et al., 2008; Chen-Harris et al., 2008). The measurement of *saccade duration* typically involves estimating the onset and offset of the saccade. Given the brief nature of saccadic movements, the accuracy of this measurement is highly sensitive to the thresholds applied to segment raw gaze data—see Section 2.

In addition to duration, *saccade count* and *saccade rate*—or *saccade frequency*—are widely used metrics to characterize saccadic sequences. Generally, *saccade frequency* tends to decrease with increasing task difficulty (Nakayama et al., 2002) or under conditions of fatigue (Van Orden et al., 2000). Like *saccade duration*, *saccade count* is a simple and robust measure commonly employed in studies that investigate cognitive processes such as reading or scene perception (Inhoff and Radach, 1998). Furthermore, deviations from typical saccadic temporal characteristics, such as prolonged *saccade duration*, can serve as early indicators of neural disorders (Ramat et al., 2007).

In experimental paradigms that involve target-directed saccades, the temporal aspect of saccadic movements is frequently examined using *saccadic latency*, which is the time delay between stimulus onset and saccade initiation. For any given target, while saccade duration, velocity, and amplitude tend to remain relatively consistent, latency is notably variable across trials, ranging from 100 to 1,000 milliseconds (Liversedge et al., 2011). The distribution of *saccadic latency* is generally skewed toward shorter latencies, with a long tail representing longer latencies. Additionally, the distribution is often unimodal, although a second peak—referred to as *express saccades*—can sometimes appear, representing shorter saccadic responses (Fischer and Weber, 1993).

The mean *saccade latency* is typically used to describe the central tendency of reaction times, while the standard deviation is used to assess variability (Whelan, 2008). However, since the latency distribution is not Gaussian, these statistics may not fully capture the nature of the distribution. As a result, more robust statistical measures, such as the median or quantile estimators, are increasingly adopted to describe saccadic latency distributions more accurately (Vullings, 2018). In clinical contexts, saccadic latency distributions have shown promise as biomarkers for various neurological conditions. For instance, Michell et al. (2006) demonstrated that saccadic latency could be used as a diagnostic marker for Parkinson’s disease, highlighting its potential utility in clinical assessments of cognitive and motor dysfunctions.

#### 3.2.2 Amplitude features

Describing saccade morphology is essential for a comprehensive understanding of eye movement dynamics. Among the various morphological features, *saccade amplitude* serves as a fundamental and easily accessible descriptor that reflects the distance the eye travels during a saccadic movement. It is typically calculated as the spatial distance between the starting and ending points of each identified saccade sequence. Alternatively, to model the non-linearity of saccade trajectory, the *traveled distance* can be computed by summing the distances between consecutive saccadic data samples within a saccade sequence. Lastly, *saccade efficiency*, derived as the ratio of saccadic amplitude to the total distance traveled, is often used to quantify the complexity and non-linearity of the saccadic trajectory. This metric provides insight into the degree to which the eye movement follows a straight path versus a more convoluted or inefficient trajectory.

*Saccade amplitude* is highly context-dependent, varying according to the task and visual environment. For example, in reading tasks, saccades are typically constrained to around 2 degrees of visual angle horizontally (Rayner et al., 2012). In contrast, during scene perception, the average *saccade amplitude* increases with the size of the visual stimulus, reflecting the broader spatial search required to process larger or more complex images (von Wartburg et al., 2007). Cognitive factors also influence *saccade amplitude*, with increases in task difficulty often leading to a decrease in the amplitude of saccadic movements. Phillips and Edelman (2008) demonstrated that variability in performance during visual scanning tasks was related to oculomotor variables such as amplitude, with smaller saccades indicating a reduced perceptual span. Similarly, May et al. (1990) provided evidence that this metric could serve as an indicator of cognitive workload, with smaller amplitudes reflecting greater cognitive demands. It should also be mentioned that *saccade amplitude* is closely related to its duration and peak velocity through the *main sequence* relationship—see Section 3.2.7 for further details. These oculomotor characteristics—amplitude, duration, and peak velocity—are often analyzed together as they provide complementary insights into the saccadic process.

When viewers are instructed to follow a visual target, the *saccadic gain*—the ratio between the amplitude of the saccade performed and the amplitude of the target displacement—becomes a critical measure. *Saccadic gain* is particularly useful in assessing saccadic dysmetria, a condition characterized by errors in saccade accuracy. In neurological studies, saccadic dysmetria is often investigated to identify impairments in saccadic control. For instance, in overshoot dysmetria, the saccade initially overshoots the target, requiring a corrective saccade in the opposite direction. While overshoots can occur in healthy individuals, they typically reduce over time as the oculomotor system adjusts to the target location. Persistent overshooting, however, is indicative of a cerebellar lesion (Selhorst et al., 1976; Ritchie, 1976). Conversely, undershoot dysmetria occurs when the initial saccade is too small, and a corrective saccade is required to bring the eye to the target. Significant undershooting is often associated with basal ganglia disorders, such as Parkinson’s disease (MacAskill et al., 2002) or progressive supranuclear palsy (Troost and Daroff, 1977).

More intriguingly, saccadic dysmetria—particularly hypometric saccades—has been proposed as a potential objective biomarker for neurodegenerative diseases. Abnormally hypometric saccades, along with other eye movement deficits, have shown promise as early indicators of conditions like Alzheimer’s disease, making them valuable targets for early diagnosis (Fletcher and Sharpe, 1986; Cerquera-Jaramillo et al., 2018). This highlights the importance of saccade morphology not only for understanding normal visual behavior but also as a potential tool for identifying and monitoring the progression of neurological disorders.

#### 3.2.3 Direction and curvature

The direction of a saccadic trajectory—or sequence of saccades—provides a crucial descriptive measure of eye movements. This direction is typically quantified as the angle, measured in degrees or radians, between the horizontal axis and the line connecting the starting and ending points of the saccade. For instance, Walker et al. (2006) employed *saccadic direction* to examine the effects of target predictability, while Foulsham et al. (2008) explored the *horizon bias* during natural scene viewing, revealing a prevalent tendency for horizontal saccades. More recently, studies have employed *saccadic direction* to classify task-specific gaze patterns, offering valuable insights for designing effective learning strategies (Mozaffari et al., 2020).

However, simple metrics such as amplitude, efficiency—as discussed in Section 3.2.2 — and direction alone are insufficient for fully capturing the complexity and non-linearity of saccadic trajectories. To address this gap, several additional features have been developed to better characterize the curvature of saccadic movements (Ludwig and Gilchrist, 2002).

One such metric is *initial deviation*, which measures the angle between the initial direction of the saccade—computed after a fixed number of time samples, *e.g.*, 20 milliseconds (Van Gisbergen et al., 1987) — and the overall direction of the saccade at its endpoint. A limitation of this method is that it assigns varying curvature values to saccades with identical trajectories but different velocities, because it relies on a fixed time interval. Another common metric is *maximum curvature*, defined as the greatest perpendicular distance between a point on the saccadic trajectory and the straight line connecting the starting and ending points of the saccade (Smit and Van Gisbergen, 1990). Although widely used, this approach has limitations, as it relies on a single point to represent the curvature of a trajectory. This can be especially problematic for double-curved saccades, where the trajectory may involve multiple directional changes (Ludwig and Gilchrist, 2002).

**FIGURE 4:**
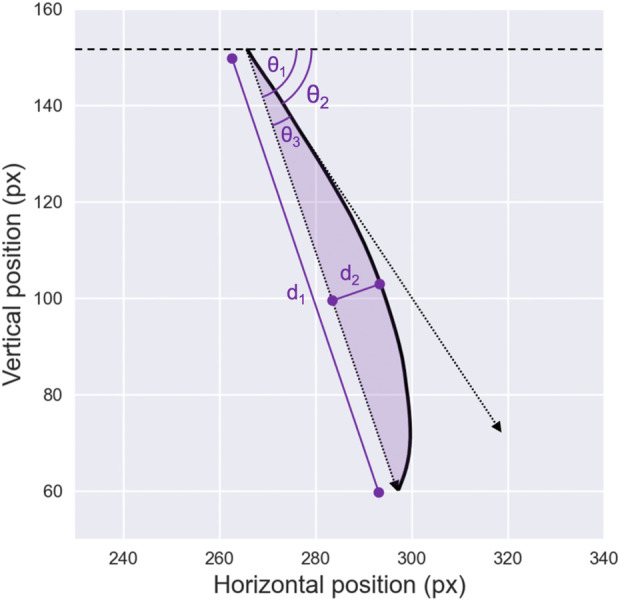
Saccade Direction and Curvature. Illustration of various metrics used to describe saccade non-linearity in the literature. The line connecting the starting point and the endpoint of the saccade, with amplitude $d_{1}$ , defines the overall saccade direction, denoted as $\theta_{1}$ . The initial direction of the saccade, denoted $\theta_{2}$ , is calculated after a fixed number of data points. From these two directions, the initial deviation of the saccade, denoted $\theta_{3}$ , can be derived. Additionally, the figure highlights the maximum curvature, represented by $d_{2}$ , and the area of curvature, indicated by the purple shaded region.

To address these shortcomings, the *area curvature* metric has emerged as a more robust and popular approach, as it incorporates the entire trajectory of the saccadic eye movement (Walker et al., 2006). This metric is typically calculated by evaluating the area beneath the curve formed by the sampled trajectory, relative to the direct distance between the starting and ending points of the saccade. The curvature metrics discussed so far are illustrated in Figure 4. Additionally, Ludwig and Gilchrist (2002) proposed deriving saccade curvature directly from second- and third-order polynomial fits. Like the *area curvature* approach, this method uses the full set of samples from a given saccade, which enhances its robustness by making it less sensitive to sampling noise.

To investigate the inherent tendency for curvature observed in saccadic movements—particularly prominent in oblique saccades (Viviani and Swensson, 1982) — early research primarily focused on target location and the type of saccade being performed (Viviani, 1977; Smit and Van Gisbergen, 1990). More recent studies, however, have shown that both the direction and magnitude of saccadic curvature can be modulated by a variety of factors. Notably, strong correlations have been observed between saccade curvature and the modulation of eye movements by distractors. For example, Doyle and Walker (2001) found that both reflexive and voluntary saccades tended to curve away from irrelevant distractor stimuli when a target was presented. Similarly, Sheliga et al. (1997), Sheliga et al. (1995) demonstrated that saccades deviated from a previously attended location. These variations in saccadic trajectory have been attributed to antagonistic interactions between different populations of neurons in the superior colliculus, which help resolve conflicts caused by competing targets in the vicinity at the onset of movement (McPeek et al., 2003).

#### 3.2.4 Velocity features

**FIGURE 5:**
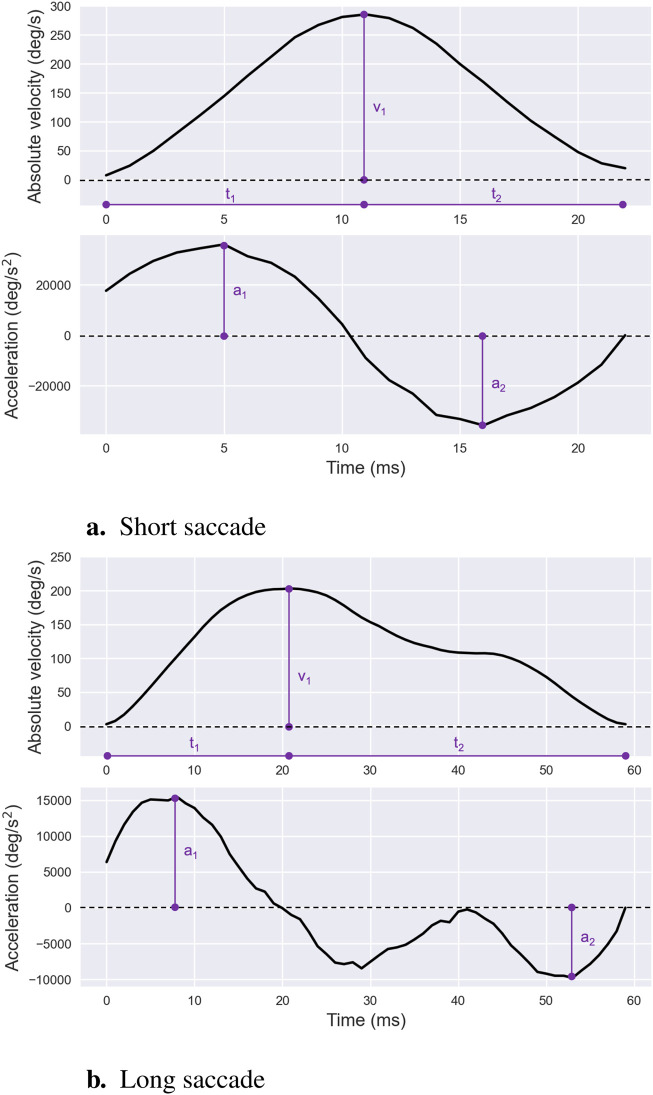
Saccade Velocity and Acceleration Profiles. Examples of saccade velocity and acceleration profiles for short — **(a)** — and long — **(b)** —- saccades, illustrating differences in peak values and overall shapes. For both types of saccades, the peak velocity is denoted as $v_{1}$ , the peak acceleration as $a_{1}$ , and the peak deceleration as $a_{2}$ . Additionally, the duration of the acceleration phase is represented by $t_{1}$ , while the duration of the deceleration phase is denoted by $t_{2}$ .

The velocity waveform of a saccade is generally described as symmetrical with comparable durations for the acceleration and deceleration phases—Figure 5a. Peak saccadic velocity, the maximum speed attained during a saccade, typically coincides with the cessation of the neural signal pulse and aligns with the point of maximum firing rate of burst neurons within the pontine reticular formation that project to oculomotor neurons (Galley, 1989; Leigh and Zee, 2015). It is noteworthy that average and peak saccadic velocities are frequently analyzed together due to their strong correlation. Their absolute values generally exhibit a consistent ratio of approximately 1:2 , a relationship commonly referred to as the *Q ratio*. This ratio remains relatively stable across various saccadic amplitudes, underscoring its reliability as a metric for characterizing saccadic dynamics (Harwood et al., 1999; Garbutt et al., 2003).

More specifically, *saccade mean velocity* is regarded as a reliable metric for assessing the velocity of small saccades, particularly those with symmetrical velocity waveforms. The properties of saccadic velocity have been thoroughly investigated across numerous fields and clinical applications (Di Stasi et al., 2013). Early research observed that external factors such as alcohol, drugs, and fatigue lead to reductions in saccadic velocity (Dodge and Benedict, 1915; Miles, 1929), a phenomenon attributed to diminished central nervous system activation. More recently, studies have highlighted saccadic velocity as a marker for fluctuations in sympathetic nervous system activity (Di Stasi et al., 2013), variations in the intrinsic value of visual stimuli (Xu-Wilson et al., 2009), and the effects of task experience on oculomotor control (Xu-McGregor and Stern, 1996). Clinically, abnormally low saccadic velocities—commonly termed *slow saccades*—are symptomatic of midbrain disorders such as progressive supranuclear palsy, spinocerebellar ataxia type 2, and various cerebellar pathologies (Jensen et al., 2019).

While mean velocity provides a useful summary metric, it becomes less effective for saccades larger than 10°, which often exhibit asymmetric velocity profiles—Figure 5b. For such larger saccades, *saccade peak velocity* is typically preferred as it reflects the highest firing rates of burst neurons driving the movement (Galley, 1989). Unlike mean velocity, peak velocity has computational advantages: it remains consistent regardless of segmentation thresholds—see Section 2 for further details—making it robust to variations in how sharply a saccade terminates during its final phase.

Several methodological considerations are important when calculating velocity features, particularly for saccades, though these principles extend to other canonical gaze movements as well. The simplest and most common method calculates velocity by applying a two-point central difference algorithm to the eye position signal (Schmidt et al., 1979). However, this straightforward approach has significant drawbacks. First, the numerical derivative is inherently highly sensitive to noise. Depending on the specific eye-tracking device, characterizing and removing measurement noise can be challenging or even infeasible. While filtering techniques can mitigate noise, they may inadvertently alter velocity estimates, particularly the crucial peak velocity. Second, this method is strongly influenced by sampling frequency. Since *saccade peak velocity* typically occurs between recorded samples, devices with low sampling rates often underestimate this key measure.

To address these limitations, more sophisticated and robust methods have been developed. These include the eight-point central difference derivative algorithm (Inchingolo and Spanio, 1985; Federighi et al., 2011), which enhances noise resilience, as well as velocity profile fitting using gamma functions (Smit et al., 1987), and saccade trajectory curve fitting using sigmoid functions (Gibaldi and Sabatini, 2021), both of which provide refined estimates by leveraging model-based approaches. These advanced techniques are robust against noise and sampling artifacts, enabling accurate velocity estimation even when using low-cost, low-sampling-rate eye trackers. This compatibility with accessible technologies broadens the utility of such methods for a wide range of research and practical applications.

#### 3.2.5 Acceleration features

To effectively quantify saccade acceleration characteristics, several metrics can be derived from the acceleration profile. As such, *saccade peak acceleration* is defined as the maximum absolute value of acceleration during the acceleration phase, which spans the interval from saccade onset to *saccade peak velocity*. Conversely, *saccade peak deceleration* represents the maximum absolute value of acceleration during the deceleration phase, occurring from peak velocity to saccade termination.

An additional metric of interest is the *acceleration/deceleration ratio*, computed as the ratio of the duration of the acceleration phase to that of the deceleration phase. This ratio reflects the skewness of the velocity profile. As expected, it tends to approximate one for small saccades but decreases as saccade amplitude increases. Finally, *saccade skewness* can be directly quantified through curve fitting, typically using a gamma function applied to the velocity profile. The resulting shape parameter provides a reliable estimate of skewness (Chen et al., 2002).

As briefly discussed in Section 3.2.4, the acceleration and deceleration characteristics of saccades vary markedly with saccade amplitude. Specifically, larger saccades exhibit left-skewed velocity profiles, where the acceleration phase constitutes roughly one-third of the total saccade duration (Baloh et al., 1975; Lin et al., 2004). This asymmetry correlates strongly with both saccade amplitude and, even more so, its duration (Van Opstal and Van Gisbergen, 1987). While the duration of the deceleration phase increases with saccade amplitude and duration, the duration of the acceleration phase remains relatively constant (Becker, 1991).

The asymmetry in saccade velocity profiles, as well as its relationship with saccade duration, has been consistently observed and documented over several decades. However, the physiological significance and underlying mechanisms of this phenomenon remain unclear, with no definitive hypothesis currently available in the literature. Research suggests that saccade acceleration characteristics may be subject to modification through motor learning processes (Collins et al., 2008). Furthermore, these characteristics have been linked to neurodevelopmental conditions, such as autism spectrum disorder, where abnormal acceleration and deceleration profiles have been observed (Schmitt et al., 2014). These findings highlight the potential for saccade dynamics to serve as biomarkers for both cognitive and neurological assessments.

#### 3.2.6 Saccadic ratios

Various ratios derived from saccadic characteristics have been extensively studied, revealing valuable insights into the interconnections between oculomotor mechanisms. For instance, Garbutt et al. (2003) identified abnormally high *peak velocity-to-mean velocity* ratios in saccadic trajectories recorded from patients with progressive supranuclear palsy. This anomaly suggested that these movements might not be purely saccadic but rather comprise a sequence of small-amplitude saccades.

In healthy individuals, saccadic ratios have been shown to reflect low-level idiosyncrasies. For example, these ratios have been employed as biometric features for individual identification among other eye-movement metrics (Rigas and Komogortsev, 2016). Extending this analysis to higher cognitive functions, Gupta and Routray (2012) demonstrated a significant correlation between the *peak velocity-to-duration* ratio and human alertness, suggesting its utility for vigilance monitoring. These findings underscore the potential of saccadic ratios as versatile markers, ranging from physiological baselines to cognitive states.

Shifting focus to broader measures of eye movement dynamics, the *saccade-fixation* ratio, introduced by Goldberg and Kotval (1999), highlights the balance between exploratory behavior—searching—and cognitive processing—information extraction. A higher value for this ratio reflects increased searching relative to processing. This metric has been used in comparative studies of different layouts or visual representations. Both the total *fixation-to-saccade duration* ratio and the average *fixation-to-saccade duration* ratio per occurrence can be derived from this measure. These simple yet powerful metrics have been employed in diverse experimental contexts to assess attention and cognitive information processing levels (Bhoir et al., 2015; Berges et al., 2023).

Finally, we mention the *K coefficient* introduced by Krejtz et al. (2016a), Krejtz et al., (2017). This metric has emerged as an extension of the *saccade-fixation ratio* and is inherently linked to scanpath analysis. As such, it will be described in greater detail in the corresponding article of this review series.

#### 3.2.7 Main sequence

**FIGURE 6:**
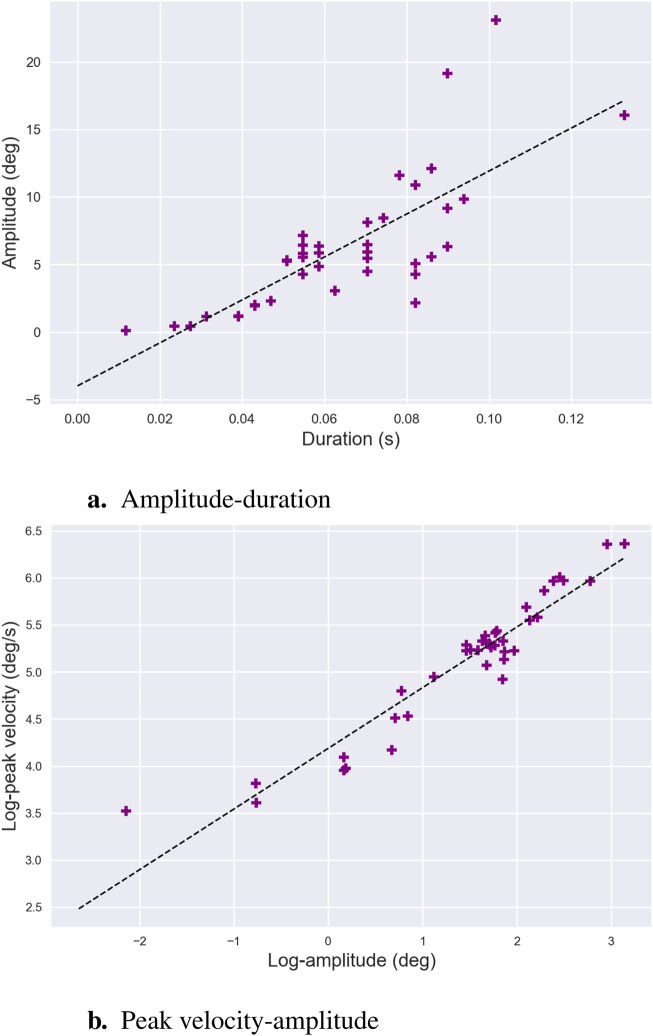
Main Sequence. Main-sequence relationships for saccades, along with the respective linear regression fits, are shown for amplitude-duration **(a)** and the logarithms of peak velocity-amplitude **(b)**. Each colored dot represents a saccade from a set performed by the same individual during a reading task. The data emphasize the linear relationship between the logarithms of amplitude and peak velocity for saccades of moderate amplitude. While the amplitude-duration relationship is well-established in the literature, its experimental clarity appears to be less consistent.

The term *main sequence* describes a consistent relationship between three fundamental saccadic parameters: amplitude, duration, and velocity (Bahill et al., 1975). Specifically, the relationship between saccadic peak velocity and amplitude demonstrates three key trends: (i) a roughly linear increase for small saccades—up to 5−−10 degrees — (ii) an inflection point between 10 and 20°, and (iii) a plateau where peak velocity saturates for larger saccades (Gibaldi and Sabatini, 2021). This stereotypical behavior is thought to result from an optimization process that improves visual performance amidst internal noise and peripheral visual uncertainty (Harris and Wolpert, 2006; Saeb et al., 2011; van Opstal and Goossens, 2008). Additionally, the *main sequence* exhibits a linear relationship between saccade duration and amplitude for saccades up to approximately 80° (Baloh et al., 1975), as shown in Figure 6A. However, most naturally occurring saccades are confined to a range of about 30° in the absence of head movement (Lebedev et al., 1996).

The *main sequence* is widely employed in clinical research as a diagnostic tool to evaluate the integrity of the saccadic system. Deviations from its expected patterns and abnormalities in saccadic behavior are indicative of various neurological and ocular conditions, including palsy of extraocular muscles (Metz et al., 1970; Garbutt et al., 2003), myasthenia gravis (Yee et al., 1976), cerebellar disorders (Selhorst et al., 1976), and multiple sclerosis (Frohman et al., 2002; Bijvank et al., 2019). Recent work by Guadron et al. (2023) further highlighted the diagnostic relevance of the *main sequence* by examining patients with central and peripheral retinal defects. Their findings revealed that the characteristic relationships between saccadic parameters were most disrupted when targets were located within the subjects’ blind fields. This disruption underscores the critical role of visual input in planning saccadic kinematics, reinforcing the *main sequence* as a valuable lens through which the interplay between sensory input and motor control can be assessed.

Despite its widespread utility, there remains no universal consensus on the best mathematical model to describe the *main sequence*, particularly the non-linear relationship between peak velocity and saccade amplitude. Early work adopted power-law models to capture the non-linear growth of peak velocity with amplitude (Yarbus and Yarbus, 1967; Baloh et al., 1975; Lebedev et al., 1996). These models have proven useful for detecting performance deficits in clinical settings (Garbutt et al., 2003). For larger saccades, 15−−20 degrees and beyond, where the maximum velocity saturates, exponential-based models have gained traction. First proposed by Bahill et al. (1975), these models have been extensively utilized in both research and clinical diagnostics (Ramat et al., 2007; Federighi et al., 2017) and remain popular for their accuracy and applicability in recent studies (Leigh and Zee, 2015). Alternatively, logarithmic transformations allow the main sequence to be expressed as linear for saccades within the 1−−15 degree range (Bahill et al., 1975; 1981), as illustrated in Figure 6b. This approach simplifies analysis while preserving the relationship’s fundamental trends.

In pursuit of greater robustness, alternative approaches have explored simpler models. For example, square-root models have been proposed to enhance the reliability of *main sequence* estimation (Lebedev et al., 1996). These models demonstrate strong generalization and repeatability, as highlighted in a recent review by Gibaldi and Sabatini (2021). Despite their simplicity, square-root models effectively capture the main sequence’s three primary trends when applied to saccades larger than 1°—a threshold that aligns with the typical amplitude range of microsaccades (Martinez-Conde et al., 2009). In conclusion, while multiple modeling approaches exist, the main sequence remains a foundational tool for understanding saccadic dynamics, with applications ranging from clinical diagnostics to explorations of the fundamental mechanisms underlying oculomotor control.

### 3.3 Smooth pursuit measures

Smooth pursuits represent another type of eye movement from which valuable metrics can be extracted. In natural scene viewing conditions, smooth pursuits occur alongside fixations and saccades to track moving objects within the field of view. To isolate these pursuit sequences, algorithms outlined in Section 2.2 must first be applied. In real-world scenarios, targets often move unpredictably, changing speed and direction rapidly. Such stimuli are rarely used in laboratory settings, as the performance of the smooth pursuit system is limited under these conditions, often resulting in interfering saccades that complicate the analysis.

In controlled experimental conditions, smooth pursuit tasks typically require the viewer to follow targets moving horizontally or vertically at a fixed frequency, back and forth. Two common types of stimuli used in these protocols are triangular and sinusoidal motion profiles. Triangular stimuli move the target at a constant velocity in one direction before abruptly reversing direction, forming a *triangle* in position-time space. This constant-velocity motion allows researchers to precisely measure the pursuit system’s ability to maintain a steady eye velocity and to detect *catch-up* saccades when the eye lags behind the target. In contrast, sinusoidal stimuli move the target in a smooth, oscillating pattern where velocity continuously varies, peaking at mid-path and slowing near the reversal points. Sinusoidal motion more closely mimics naturalistic motion and tests the pursuit system’s ability to adapt to continuously changing velocities. In these experimental setups, it is typically assumed that the oculomotor signal reflects primarily smooth pursuit eye movements, along with any catch-up saccades, without the inclusion of fixation sequences. The pursuit system is expected to generate smooth, coordinated eye movements that closely follow the target’s trajectory, minimizing interruptions from fixational pauses.

#### 3.3.1 Temporal and velocity features

The analysis of smooth pursuit eye movements typically starts with the estimation of fundamental descriptors, such as *pursuit duration*, *pursuit count*, and *pursuit rate*—or *pursuit frequency*. However, interpreting these metrics is not as straightforward as it might initially appear. This complexity arises primarily from the influence of *catch-up saccades*, which are corrective eye movements that compensate for discrepancies between the target’s position and the smooth pursuit response. These saccades interrupt smooth pursuit sequences, effectively shortening their duration while increasing the overall *pursuit frequency*.

More specifically, *catch-up saccades* are rapid eye movements that occur during smooth pursuit when the eye falls behind the target. They help correct the eye’s position by quickly redirecting the gaze to the moving target. These saccades occur when the smooth pursuit mechanism, which is responsible for maintaining the eye’s tracking of a moving object, is unable to keep up with sudden changes in the target’s velocity or direction. Catch-up saccades are particularly common when the target moves too fast for the smooth pursuit system to follow continuously or during pursuit of targets with unexpected changes in velocity or direction (Boman and Hotson, 1992). Instead of maintaining a smooth motion, the eyes make these corrective jumps to *catch up* with the target, thus ensuring the target stays within the central vision. Additionally, their occurrence is modulated by factors such as target properties (Heinen et al., 2016) and clinical conditions, including schizophrenia and affective disorders (Abel et al., 1991).

Characterizing the velocity profile of smooth pursuit typically involves measurements of *pursuit mean velocity* and *pursuit peak velocity*. Smooth pursuit velocities are generally modest, ranging between 15 and 30° per second (Meyer et al., 1985; Zuber et al., 1968; Ettinger et al., 2003; Klein and Ettinger, 2019), significantly lower than saccadic velocities. However, trained observers or tasks involving accelerating stimuli can elicit higher peak velocities. For instance, Barmack (1970) reported peak pursuit velocities of up to 100° per second during acceleration tasks. In humans, peak eye velocity typically occurs between 200 and 300 milliseconds after pursuit onset when following targets moving at velocities up to 30° per second (Robinson et al., 1986).

Importantly, the velocity profile is closely linked to temporal characteristics: as stimulus velocity increases, the frequency of *catch-up saccades* also rises to correct for larger retinal offsets. A valuable descriptor for exploring this relation between velocity and compensation mechanisms is *eye crossing time*, defined as the duration required for the eye to align with the target at constant velocity. De Brouwer et al. (2002) demonstrated that catch-up saccades are initiated when the eye crossing time reaches the saccade zone, indicating that smooth acceleration alone is insufficient for target capture.

However, simple spatio-temporal features such as *pursuit mean velocity* and *pursuit duration* do not fully capture the complexity of smooth pursuit dynamics. Smooth pursuit consists of two distinct phases: *open-loop* and *closed-loop*. In the open-loop phase, the eye’s movement is primarily driven by the initial target presentation, with little to no influence from the retinal image changes caused by the eye movement. In contrast, during the closed-loop phase, the eye continuously adjusts to changes in the retinal image that result from its own movements, maintaining the pursuit of the target. In the following Sections 3.3.2, 3.3.3, we will introduce methods to quantify the initiation and maintenance of pursuit, respectively.

#### 3.3.2 Smooth pursuit latency and acceleration

In this section, we introduce two classes of features used to characterize the pursuit initiation phase, namely, *pursuit latency* and *pursuit acceleration*. In target pursuit paradigms, *pursuit latency*—or *pursuit onset*—is commonly defined as the delay between the initiation of target motion and the start of ocular pursuit. The onset of smooth pursuit is typically calculated as the intersection point between two regression lines (Carl and Gellman, 1987). The first line represents the *pre-response baseline*, which fits the velocity signal during a time window from 100 milliseconds before target motion onset to 80 milliseconds after it begins. This baseline duration may vary depending on the experimental setup, particularly when anticipation of the target motion is expected (De Hemptinne et al., 2006). The second regression line fits the *pursuit initiation* velocity signal, typically recorded over a 50 milliseconds window after the pre-response baseline. This duration may differ across studies, often beginning at the first time point when eye velocity exceeds three to 4 standard deviations of the baseline velocity measures (Krauzlis and Miles, 1996).

Pursuit typically exhibits much shorter latency than saccades, with *pursuit latency* ranging from 100 to 125 milliseconds, compared to 200–250 milliseconds for saccades (Krauzlis, 2004). In experimental conditions involving anticipation, pursuit latency can be reduced to zero or even become negative, especially when pursuit begins before the target motion, such as when the direction and velocity of the stimulus are highly predictable (Burke and Barnes, 2006; De Hemptinne et al., 2006). Spering and Gegenfurtner (2007) further demonstrated that *pursuit latency* is influenced by the surrounding visual context, particularly by contrast and distracting motion orientation. They found that latency decreases when the context moves in the same direction as the target, while a rapidly moving context in the opposite direction tends to *pull* the eyes back, delaying pursuit onset. Additionally, higher contrast enhances the effect of co-linear drifting context motion, further reducing the latency before the pursuit begins.

In addition to latency, pursuit initiation is often examined through *pursuit initial acceleration* (Kao and Morrow, 1994). This is typically calculated as the mean second-order position derivative of the saccade-free component extracted from the tracking response within the first 100 milliseconds following pursuit onset. During this initial phase, acceleration continues until the eye velocity matches that of the target. The *pursuit initial peak acceleration* can also be assessed during this period. The first 20–30 milliseconds of eye acceleration show a modest increase with target velocity (Tychsen and Lisberger, 1986). However, between 60 and 80 milliseconds after pursuit onset, eye acceleration becomes much more strongly modulated by target velocity, and is also influenced by the eccentricity of the initial eye position (Fukushima et al., 2013).

Furthermore, like latency, the *pursuit initial acceleration* is significantly influenced by expectations regarding the target’s trajectory (Kao and Morrow, 1994). Prior knowledge of the target’s movement—not only from its motion history but also from static visual cues—profoundly affects eye movements during pursuit initiation (Kao and Morrow, 1994; Ladda et al., 2007). Notably, Ladda et al. (2007) found that cue-induced acceleration during smooth pursuit increases quadratically with target velocity. This behavior aligns with the velocity scaling predicted by the *two-thirds power law*, a natural principle of biological motion (Lacquaniti et al., 1983).

#### 3.3.3 Pursuit gain and accuracy

Smooth *pursuit gain* refers to the ratio of the eye’s mean velocity to the target’s mean velocity during a pursuit segment, typically under constant target velocity conditions, often referred to as *triangular stimuli*. This metric is generally assessed around 500–1,000 milliseconds after pursuit onset, during the *pursuit maintenance* phase, and serves as a measure of pursuit performance. During pursuit initiation, which occurs within the first 50–100 milliseconds after the target starts moving, pursuit gain is primarily controlled by visual motion (Rashbass, 1961). However, in the *pursuit maintenance* phase, the gain is influenced by a combination of visual feedback regarding performance quality and internal cues, such as anticipation and prediction of target velocity (Lencer and Trillenberg, 2008). This stable regime facilitates a more accurate assessment of performance compared to the more transient initiation phase. Typically, smooth pursuit gain is lower than 1, indicating that the eye lags behind the target, and it tends to decrease as target velocity increases (Zackon and Sharpe, 1987).

In sinusoidal stimulation paradigms, the smooth pursuit response is usually described by two key characteristics: *pursuit velocity phase* and *pursuit velocity gain* (Accardo et al., 1995). These values are derived by fitting the eye velocity data with a trigonometric curve for each experimental pursuit sequence. The *pursuit velocity gain* is then computed as the ratio of the peak velocity of the best-fitting curve to the peak velocity of the target’s trajectory. Similarly, the *pursuit velocity phase* is computed as the phase difference between the best-fitting velocity curve and the target’s velocity profile. Note that *overall gain* is also widely used in the literature, calculated as the ratio of eye velocity to target velocity (Churchland and Lisberger, 2002).

Smooth pursuit is often conceptualized as a negative feedback control system in which smooth eye acceleration works to eliminate retinal motion by matching the eye velocity to the target velocity. However, substantial evidence suggests that smooth pursuit gain is modulated by an *on-line* gain control mechanism, which implies distinct visual-motor gain processing during pursuit and fixation (Robinson, 1965; Churchland and Lisberger, 2002). It is now widely accepted that visual inputs are not the sole mediators of smooth pursuit. Higher-order brain functions, such as attention, have been shown to play a significant role in pursuit gain and performance, though their effects have been debated (Březinová and Kendell, 1977; Acker and Toone, 1978; Kathmann et al., 1999; Van Gelder et al., 1995). Studies suggest that attention is crucial for pursuit performance (Van Donkelaar and Drew, 2002), but Stubbs et al. (2018) demonstrated that while increased attentional demands do not alter smooth pursuit gain, they do improve its consistency, as long as attention remains focused on the target.

Furthermore, smooth pursuit performance can be influenced by a trade-off between perceptual discrimination and pursuit efficiency. Specifically, when a perceptual discrimination task involves objects moving at a different velocity from the pursuit target, the ability to maintain smooth pursuit is compromised (Khurana and Kowler, 1987). More recently, Kerzel et al. (2009) or Souto and Kerzel (2014) have further confirmed this interdependence between target selection for pursuit and perceptual processing. This interaction is generally understood as reflecting a shared, limited resource that is required for both steady-state smooth pursuit and perceptual tasks (Stolte et al., 2023).

Finally, smooth pursuit gain has become a crucial measure in neuro-pathological research. For example, a review by Franco et al. (2014) highlighted studies showing that individuals diagnosed with schizophrenia often exhibit lower smooth pursuit gain. Smooth pursuit performance is also a valuable tool in assessing sensorimotor development in preadolescence and adolescence. Horizontal smooth pursuit typically matures by age 7 (Ingster-Moati et al., 2009), while vertical smooth pursuit does not reach maturity until late adolescence (Katsanis et al., 1998). This asymmetry between horizontal and vertical pursuit is due to the involvement of different brain structures in controlling these movements (Collewijn and Tamminga, 1984; Grönqvist et al., 2006), with significant clinical implications. For instance, Robert et al. (2014) demonstrated that children with developmental coordination disorder often exhibit impaired vertical pursuit performance, indicating delayed maturation of the pursuit system in this population.

## 4 Signal analysis

In this section, we review time series analysis methods for the study of ocular behavior. Compared to traditional neurophysiological approaches, these methods are underexplored but offer a robust framework for analyzing eye movements as a cohesive, dynamic system. In contrast to neurophysiological methods, which focus on specific neural circuits associated with individual eye movement types, time series approaches capture the temporal and structural patterns of eye behavior across contexts. Table 4 summarizes the metrics and algorithms discussed, describes each method and the required input formats, and provides key literature references to facilitate implementation.

**TABLE 4:** Signal-based features.

Feature name	Description	References
Periodogram	Given a raw gaze signal, estimates power spectral density	McGillem and Cooper (1991)
Welch periodogram	Given a raw gaze signal, estimates power spectral density, using a Welch windowed periodogram	Welch (1967)
Cross spectral density	Given a set of raw gaze signals, estimates the cross power spectral density between pairs of signals	McGillem and Cooper (1991)
Welch cross spectral density	Given a set of raw gaze signals, estimates the cross power spectral density between pairs of signals, according to Welch’s method	McGillem and Cooper (1991)
Coherency	Given a set of raw gaze signals, estimates how strongly pairs of signals are related at specific frequencies	Bendat and Piersol (1986)
Mean squared displacement	Given a raw gaze signal, estimates the average squared deviation of the eye-gaze position from a reference position over time	Herrmann et al. (2017)
Displacement auto-correlation function	Given a raw gaze signal, estimates the degree of similarity between the gaze signal and a lagged version of itself over successive time intervals	Herrmann et al. (2017)
Detrended fluctuation analysis	Given a raw gaze signal, estimates long-range correlations and scaling behavior by analyzing signal fluctuations over different time scales	Wang and Cong (2015)
Persistence size	Given a raw gaze signal, estimates the entropy of the size of the holes in the persistence diagram obtained from gaze signal	Chung et al. (2021)
Persistence robustness	Given a raw gaze signal, estimates the entropy of the robustness of the holes in the persistence diagram obtained from gaze signal	Chung et al. (2021)
Betti curve	Given a raw gaze signal, estimates a function evaluating the Betti numbers obtained from a persistence diagram, at different levels of filtration	Güzel and Kaygun (2023)
persistence curve	Given a raw gaze signal, estimates a function that summarizes the total persistence of topological hole of the persistence diagram, at different levels of filtration	Kachan and Onuchin (2021)
Persistence entropy	Given a raw gaze signal, estimates the Shannon entropy of the collections of topological holes lifetimes of the persistence diagram obtained from gaze signal	Kachan and Onuchin (2021)

### 4.1 Frequency variables

Section 3 described methods for characterizing eye movements, focusing on spatial and temporal attributes such as fixation locations and saccade kinematics. These approaches often neglect the dynamic processes underlying these patterns. Spectral analysis provides an alternative framework by examining the frequency content of eye movement time series, revealing oscillatory patterns that reflect underlying dynamics (Stoica and Moses, 2005).

The spectral content of gaze data is commonly analyzed using the *discrete Fourier transform* (DFT), which converts the ocular signal into a frequency-domain representation (McGillem and Cooper, 1991). The DFT decomposes the signal by correlating it with sinusoids of varying frequencies, identifying dominant rhythmic components. The *power spectral density* (PSD) complements this by quantifying the amplitude of these rhythms as a function of frequency, offering insights into the signal’s oscillatory structure. Welch’s method (Welch, 1967), a widely adopted PSD estimation technique, segments the signal into overlapping windows, applies a window function, and averages the squared DFT magnitudes across segments. This approach balances frequency resolution and statistical reliability, yielding robust PSD estimates with reduced noise.

Spectral analysis also enables comparative studies of gaze data through metrics such as cross-spectral density and signal coherence, which are valuable for analyzing eye movement behavior across experimental conditions, individuals, or species (Ko et al., 2016). *Cross-spectral density* measures the frequency-specific covariance between two signals, while *signal coherence*, derived from cross-spectral density, quantifies the consistency of phase relationships, revealing synchronized rhythmic activities. For instance, Nakayama and Shimizu (2004) used cross-spectral density to demonstrate task-related differences in the coordination of horizontal and vertical eye movement components, highlighting the influence of task difficulty. Additionally, spectral analysis has been applied to compare real and synthetic gaze data, enabling evaluation of generative models. Duchowski et al. (2016) utilized spectral analysis to distinguish experimentally recorded gaze patterns from synthetic ones, advancing insights into eye movement dynamics.

### 4.2 Stochastic variables

Directly comparing eye movement data is challenging due to the stochastic, or inherently random, nature of gaze signals, as discussed in Section 3. Modeling eye movements as random variables provides an alternative approach, uncovering physiological patterns through their statistical characteristics. A key tool, the *mean squared displacement* (MSD), tracks how gaze positions shift over time. In simple random walks, like Brownian motion with independent steps, the spread grows steadily. In complex cases, such as eye movements, the spread follows a power-law pattern, reflecting diverse neural and behavioral dynamics.

Isolated fixational eye movements, such as microsaccades and drift, are well-suited for stochastic analysis due to their structured yet random nature. Engbert and Kliegl (2004) used the MSD to reveal distinct patterns in these movements. On short time scales—tens to hundreds of milliseconds—fixational movements are persistent, following consistent directions to promote retinal shifts that prevent visual fading. On longer time scales, they become *anti-persistent*, with negatively correlated increments that facilitate maintaining gaze on the intended fixation point.

Detrended fluctuation analysis (DFA), another powerful method, quantifies long-term power-law correlations in non-stationary gaze data. Moshel et al. (2008) applied DFA to demonstrate that microsaccades enhance persistence more in horizontal than vertical fixational movements, suggesting distinct neural control mechanisms for these components (Sparks, 1986; Moschovakis, 1996). Beyond physiological studies, DFA has been used in functional research. For example, Wang and Cong (2015) employed DFA to investigate how professional experience shapes eye movement patterns in air traffic controllers, linking gaze dynamics to cognitive and task-related factors.

Finally, the MSD analysis of fixational movements exhibits oscillatory behavior over longer time scales (Herrmann et al., 2017). The displacement auto-correlation function (DACF) complements MSD by comparing a movement’s trajectory to its delayed versions, highlighting these rhythmic patterns. Such patterns suggest that drift movements are centrally controlled, potentially through time-delayed feedback mechanisms (Herrmann et al., 2017). These methods, summarized in Table 4, provide insights into the dynamic control of gaze allowing to explore additional temporal patterns.

### 4.3 Topological variables

Recent studies have applied topological data analysis (TDA) to investigate the complex patterns of eye movement trajectories. Conventional measures, such as fixation durations or saccade amplitudes, often fail to capture the broader spatial and temporal structure of gaze patterns. Pioneering works by Kachan and Onuchin (2021) and Onuchin and Kachan (2023) addressed this limitation by using TDA to extract novel features from eye movement data, demonstrating improved performance in recognition tasks on new gaze trajectory datasets. More recently, He et al. (2025) showed that spatial-temporal topological features derived from eye-tracking data can be informative for neural disorder screening, highlighting the clinical relevance of these TDA-based representations.

**FIGURE 7:**
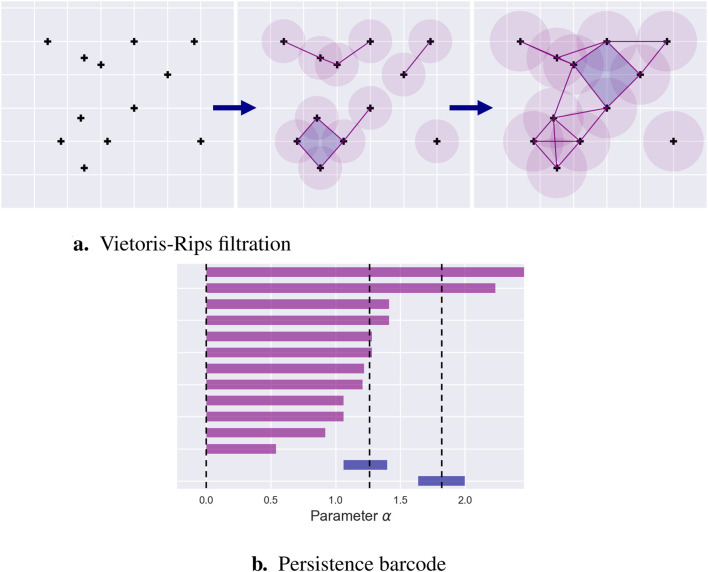
Forming Persistence Diagrams. Given a set of points—gaze data-samples—the Vietoris-Rips filtration approximates the topology of the union of the balls of radius equal to the threshold parameter α centered at each point from the dataset. The **(a)** shows, for three values of α — also represented by dotted lines in **(b)** — appearance of topological features of dimension 0 — purple lines for connected components—and dimension 1 — blue shaded areas for holes. The persistence diagram, or persistence *barcode*, plotted **(b)** of dimension 0 — purple bars—summarizes the linking of clusters while the persistence diagram of dimension 1 — blue bars—summarizes the number of topological holes between clusters, describing the complexity of clusters arrangement.

A central tool in TDA is persistent homology, which provides a way to measure the *shape* of a dataset across multiple scales. To illustrate, consider a set of eye positions represented as points in space. Persistent homology tracks the formation and disappearance of topological features, including connected clusters of points, circular arrangements forming loops, and higher-dimensional empty regions called voids. These features are identified through a process called a filtration, in which a scale parameter gradually increases. Initially, each point is separate, but as the scale grows, points that are close to each other become connected. A topological feature is said to be *born* when it first appears, for example, when two points merge into a cluster or a loop forms, and it *dies* when it disappears, such as when two clusters merge into one larger cluster or a loop is filled in. By recording the birth and death of each feature, the structural information of the dataset can be summarized in a persistence diagram, where longer-lived features typically represent meaningful structures while short-lived features correspond to noise (Carlsson, 2009; Edelsbrunner and Harer, 2022). Figure 7 illustrates this process schematically.

One common method to build topological structures is the Vietoris-Rips complex. In this approach, points in a cloud are connected if they are within a certain distance defined by the current scale parameter. Sets of points that are mutually connected form higher-dimensional shapes: a pair of points forms a line segment, three points form a filled triangle, and four points form a tetrahedron. As the scale increases, more connections are added, creating new features or merging existing ones. This gradual growth generates the birth and death events that are tracked in persistent homology.

Kachan and Onuchin (2021) proposed two TDA-based approaches for analyzing eye movements. In the first, eye positions are treated as a point cloud, ignoring timestamps, to capture spatial patterns. In the second, horizontal and vertical gaze coordinates are analyzed as separate time series to study temporal dynamics. From these representations, persistence diagrams are derived and transformed into compact features, such as the lifespan of topological features or their stability across scales. These features can be computed for Vietoris-Rips complexes or for sub-level set filtrations, which track the appearance and disappearance of features as the values of the data themselves vary, for example, along intensity or velocity thresholds. Persistence diagrams can then be vectorized into structured formats suitable for machine learning, enabling classification, clustering, or other data-driven analyses. By emphasizing shape-related properties of gaze data, TDA provides tools to capture structural patterns that traditional metrics often overlook, and as shown by He et al. (2025), these spatial-temporal topological features can also serve as biomarkers for neural disorder screening.

## 5 Discussion

The segmentation of raw gaze data into a sequence of oculomotor events remains a cornerstone of eye movement research. In this article, we have reviewed the most common segmentation algorithms—Section 2). Historically, threshold-based methods dominated the field, relying on predefined criteria such as velocity or displacement thresholds to categorize eye movements. These approaches remain widely used because of their simplicity, computational efficiency, and relatively low barrier to implementation. However, they also exhibit critical limitations: their sensitivity to parameter selection can lead to inconsistent results across laboratories, and their robustness often degrades in noisy or dynamic environments, such as mobile or low-cost eye trackers. These drawbacks highlight the need for approaches that are less dependent on arbitrary thresholds and more adaptable to variability in recording conditions.

In contrast, learning-based approaches have gained prominence by leveraging annotated datasets that encode expert knowledge of eye movement types. By training models on rich and diverse data, these methods can capture complex patterns in the gaze signal that extend beyond traditional definitions of fixations, saccades, and pursuits. For instance, they are better suited to handle ambiguous or overlapping cases, where threshold-based approaches often fail. Nevertheless, their performance is critically dependent on model architecture, hyperparameter optimization, and, above all, the quality, diversity, and size of the training datasets. A model trained on limited or biased data may perform well within a narrow domain but fail to generalize to different populations, tasks, or devices. This dependency underscores the importance of carefully curated datasets and rigorous cross-validation protocols.

To foster transparency and reproducibility in machine learning–based segmentation, detailed methodological reporting is essential. Beyond describing the general algorithmic approach, authors should provide explicit documentation of the algorithms and software packages employed, the hyperparameter configurations chosen, and the strategies used for validation. Where feasible, access to training and validation datasets should also be shared, either through open repositories or upon reasonable request. Such openness ensures that results can be replicated, facilitates the systematic refinement of models, and lowers the entry barrier for new research groups seeking to build upon existing work. Ultimately, transparent reporting practices strengthen confidence in published findings and encourage convergence toward best practices in the field.

In this regard, specialized databases are playing an increasingly central role. Resources such as the GazeBase dataset (Griffith et al., 2021) provide large and heterogeneous eye movement recordings across diverse tasks, from controlled guided stimuli designed to elicit specific movements, to goal-directed activities, and free-viewing scenarios such as reading or video watching. These datasets are indispensable for benchmarking both traditional and learning-based algorithms, enabling fair comparisons across methods, and for training models with stronger generalizability across tasks and hardware. By facilitating standardized evaluation, such databases support the transition from isolated methodological contributions toward a cumulative science of eye movement analysis. Looking ahead, the expansion of open repositories covering diverse populations, age groups, and experimental contexts will be critical for building robust segmentation algorithms with real-world applicability.

Beyond segmentation itself, this article has also reviewed the metrics derived from canonical oculomotor events—Section 3). These metrics are essential for characterizing fixations, saccades, and smooth pursuits in terms of their temporal, spatial, and kinematic properties, and for linking them to cognitive, clinical, and applied research contexts. For example, fixation duration can be tied to attentional processes, while saccade amplitude and velocity are informative about motor control and neurological function. However, meaningful cross-study comparisons are only possible if these metrics are computed in standardized ways and interpreted within a shared conceptual framework. Advancing this line of work therefore requires: (i) a unified set of definitions and formal concepts, (ii) standardized analytical pipelines that minimize methodological variability, and (iii) accessible open-source datasets and software packages that encourage reproducibility and methodological convergence. Together, these elements will harmonize computational practices, foster interdisciplinary collaboration, and ultimately improve the comparability and interpretability of findings across the diverse fields that rely on eye movement research.

It is important to stress, however, that the robustness of segmentation and derived metrics depends strongly on the hardware employed. High-speed laboratory-grade eye trackers — 500−−1000Hz — provide fine-grained temporal resolution, yielding reliable estimates of fixation stability, saccade dynamics, and pursuit gain. In these conditions, reproducibility is typically high for metrics such as RMSD or Cohen’s Kappa. By contrast, low-cost or mobile devices — 30−−120Hz — are more prone to noise and data loss, which introduces uncertainty in event boundaries. Fixations, being relatively long in duration, are somewhat resilient, although noise can still inflate false positives. Saccades, in turn, are especially vulnerable: low sampling rates may miss peak velocities or misestimate onset and offset times, leading to degraded temporal precision and event-level accuracy. These differences underscore the need for robust, hardware-agnostic metrics that remain interpretable across diverse research settings.

Looking ahead, several technological and methodological trends promise to reshape oculomotor research. The rapid adoption of VR platforms equipped with eye tracking enables exploration of gaze behavior in immersive, ecologically valid 3D contexts, where traditional eye movements interact with head and body dynamics (Adhanom et al., 2023). The growing use of mobile eye tracking is similarly expanding research far beyond lab settings, though it raises significant challenges in data quality and reproducibility (Fu et al., 2024). On the computational front, while AI and deep learning methods for event segmentation are emerging, the need for rigorous evaluation and privacy-aware implementations remains pressing—especially in VR contexts (Bozkir et al., 2023). More broadly, as Extended Reality (XR) environments integrate eye tracking with multimodal sensors, methodologies must adapt to both technological possibilities and ethical considerations (Kourtesis, 2024). Together, these advances point toward richer, more scalable, and context-sensitive analyses of oculomotor behavior.

Finally, we reviewed emerging approaches that challenge the traditional paradigm of segmentation into discrete events—Section 4. Advanced signal processing methods, such as topological data analysis (TDA), enable the study of the intrinsic structure of eye movement signals without imposing predefined categories. By focusing on patterns such as connectivity, loops, or voids in gaze trajectories, TDA captures structural properties that may be overlooked by conventional event-based frameworks. This represents a promising step toward more naturalistic analyses, particularly in contexts where boundaries between fixations, saccades, and pursuits are ambiguous or functionally irrelevant. As these methods mature, they are likely to complement existing frameworks and enrich our understanding of oculomotor control in real-world visual behavior.
